# Antibodies in serum of convalescent patients following mild COVID‐19 do not always prevent virus‐receptor binding

**DOI:** 10.1111/all.14523

**Published:** 2020-08-27

**Authors:** Pia Gattinger, Kristina Borochova, Yulia Dorofeeva, Rainer Henning, Renata Kiss, Bernhard Kratzer, Bernhard Mühl, Thomas Perkmann, Doris Trapin, Martina Trella, Paul Ettel, Inna Tulaeva, Winfried F. Pickl, Rudolf Valenta

**Affiliations:** ^1^ Department of Pathophysiology and Allergy Research Division of Immunopathology Center for Pathophysiology, Infectiology and Immunology Medical University of Vienna Vienna Austria; ^2^ Viravaxx Vienna Austria; ^3^ Institute of Immunology Center for Pathophysiology, Infectiology and Immunology Medical University of Vienna Vienna Austria; ^4^ Labors.at Vienna Austria; ^5^ Department of Laboratory Medicine Medical University of Vienna Vienna Austria; ^6^ Laboratory for Immunopathology Department of Clinical Immunology and Allergy Sechenov First Moscow State Medical University Moscow Russia; ^7^ NRC Institute of Immunology FMBA of Russia Moscow Russia; ^8^ Karl Landsteiner University of Health Sciences Krems Austria

**Keywords:** COVID‐19, immune complex, molecular interaction assay, protective antibodies, SARS‐CoV‐2, vaccine

**Abbreviations**
ACE2: angiotensin‐converting enzyme 2
COVID‐19: coronavirus disease
ELISA: enzyme‐linked immunosorbent assay
HRP: horseradish peroxidase
IgG, IgA, IgM: immunoglobulin G, A, M
MERS: middle east respiratory syndrome
OD: optical density
RBD: receptor‐binding domain
RNA: ribonucleic acid
RT‐PCR: reverse transcription polymerase chain reaction
RV: rhinovirus
S: spike protein
S1: spike protein receptor‐binding subunit
S2: spike protein membrane fusion subunit
SARS: severe acute respiratory syndrome

To the Editor,

After the appearance of first cases in Wuhan, China in December 2019, the novel human coronavirus disease, COVID‐19, has become the first coronavirus pandemic in history.^1^ On 16 July 2020, more than 13.5 million patients worldwide have been infected with the novel coronavirus, SARS‐CoV‐2, and more than 584.000 global deaths related to COVID‐19 have been reported (see: The Center for Systems Science and Engineering (CSSE) at Johns Hopkins University, Baltimore: https://gisanddata.maps.arcgis.com/apps/opsdashboard/index.html#/bda7594740fd40299423467b48e9ecf6). The first descriptions of coronaviruses date back to the 1930s when they were isolated from chickens. Originally, coronaviruses were associated with important diseases in cattle, poultry, pigs and cats. They are large, enveloped, positive‐stranded RNA viruses with round structure and long, petal‐shaped spikes protruding from their surface. Coronaviruses can be divided into three serogroups of which groups I and II have been isolated from mammals and group III from birds. Members from groups I and II (Group I: HCo‐229E, HCoV‐NL63; Group II: HCoV‐OC43, HCoV‐HKU1) have been known for decades as causes for relatively mild common colds in humans. However, in 2002, severe acute respiratory syndrome (SARS) (Group IIb) and, in 2012, Middle East respiratory syndrome (MERS) (Group IIc) were shown to be caused by the novel coronaviruses, SARS‐CoV and MERS‐CoV, respectively, which caused high death rates in up to 10% of infected people.^1^

Like SARS‐CoV, SARS‐CoV‐2 uses angiotensin‐converting enzyme 2, ACE2 on human cells as its receptor^2^ and binds to it with its receptor‐binding domain (RBD). The RBD is located in the spike protein S within S1, the receptor‐binding subunit close to the C‐terminal S2 membrane fusion subunit.^2^ The clinical course of COVID‐19 has a tri‐phasic pattern with fever, cough, fatigue in week 1, dyspnoea, lymphopenia and pneumonia in week 2 and resolution in week 3. However, in severe cases, thrombocytopenia, coagulopathy, acute kidney injury, myocardial injury, respiratory distress syndrome and deteriorating multi‐organ dysfunction can occur.^3^

Acute infection can be diagnosed by demonstrating the presence of virus‐derived nucleic acid by RT‐PCR in nasopharyngeal swabs in patients. However, there is currently no specific and effective treatment for COVID‐19. Accordingly, quarantine, social distancing and enhanced hygiene precautions are the only measures to prevent virus spread.

It has been shown that COVID‐19 patients develop SARS‐CoV‐2‐specific antibodies but it is not known if and in how many infected subjects the virus‐induced antibodies are protective.

In order to investigate whether COVID‐19 convalescent patients have developed antibodies that may protect from reinfection, we collected sera from COVID‐19 convalescent patients approximately 10 weeks after confirmation of COVID‐19 by qRT‐PCR (Table S1) (group B, n = 25, 11 females, 14 males, age range: 18‐70 years, median age 52.2) and included for control purposes sera from subjects obtained before the COVID‐19 pandemic (historic control group P, n = 24, 13 females, 11 males, age range: 18‐68 years, median age 43.2) (Table S1). The course of COVID‐19 in the PCR‐confirmed convalescent subjects (group B) was relatively mild and did not require hospitalization but the duration of COVID‐19‐related symptoms varied considerably among patients (ie from 1 to 23 days) (Table S1). COVID‐19 convalescent patients showed a quite strong and distinct IgG reactivity to S and RBD whereas no RBD‐specific IgG was found in all but one (ie P014) of the historic control sera (group P) of whom few showed some S‐specific IgG (Figure 1). IgA anti‐RBD and anti‐S responses measured in a subset of COVID‐19 convalescent patients were low and not detectable in a subset of historic controls (Figure S1, Methods in the Appendix S1). Strong S‐ and RBD‐specific IgM responses were found in convalescent patients but we found also frequent and distinct IgM responses in the historic controls (Figure 1). In this context, it must be mentioned that S and RBD contain several glycosylation sites (Figure S2) (see reference in the Appendix S1). S and RBD used in our ELISA were expressed in eukaryotic cells and hence were glycosylated which would explain the occasional and weak recognition by IgG and the more frequent recognition by IgM, an isotype frequently reacting with glycan moieties, by the presence of anti‐carbohydrate antibodies in the sera. It is therefore quite possible that anti‐glycan antibodies may give “false” positive test results when glycosylated RBD or spike proteins are used in serological assays for COVID‐19. RBD‐specific IgG levels determined by ELISA were highly correlated with SARS‐CoV‐2‐specific antibodies determined with the fully automated Siemens, Atellica IM SARS‐CoV‐2 Total (COV2T) test (see methods in Appendix S1, Figure S3A, Table S2). We also found a significant correlation of RBD‐specific IgM levels measured by ELISA and the Siemens test (Figure S3B).

**FIGURE 1:**
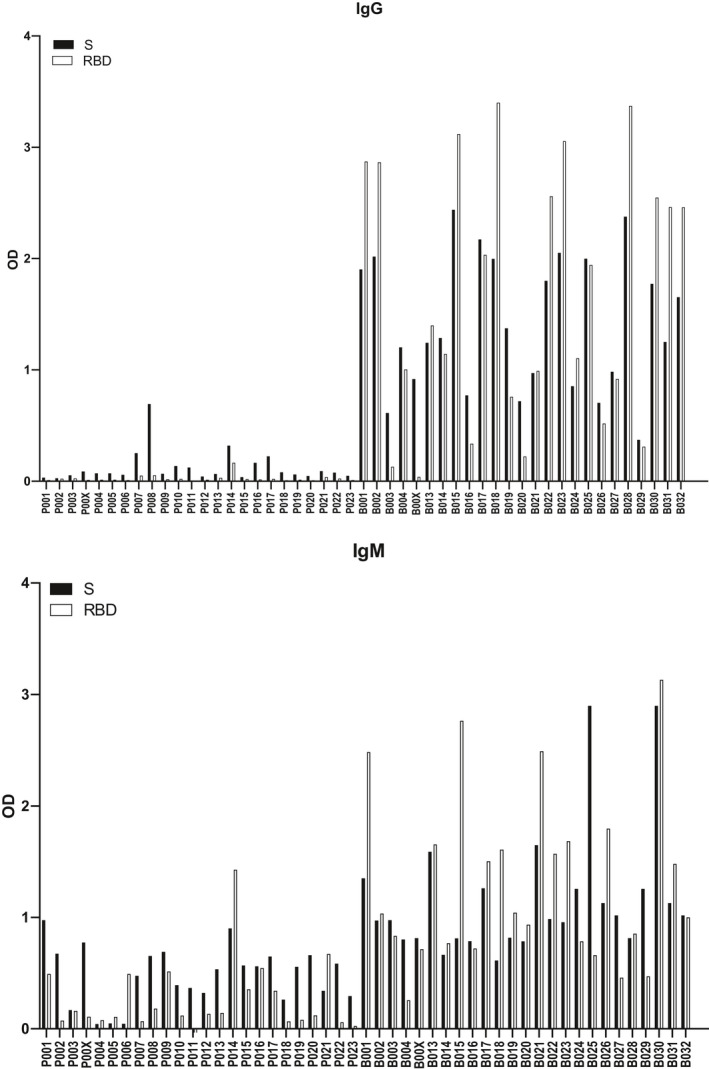
IgG (upper panel) and IgM (lower panel) reactivity (y‐axis: OD values corresponding to bound immunoglobulin) to S and RBD determined for COVID‐19 convalescent patients (group B: B001‐B032, right) and for individuals from a historic control group before the pandemic (group P: P001‐P023, left). The threshold for background has been subtracted

Receptor‐binding domain‐specific IgM responses in COVID‐19 convalescent patients were not always associated with corresponding IgG responses (Figure 1). For example, subjects B003 and B00X showed RBD‐specific IgM reactivity whereas they mounted almost no RBD‐specific IgG and subject B004 contained S‐ and RBD‐specific IgG but no specific IgM was detected (Figure 1). We found no correlation between S‐specific IgM and IgG responses and a significant correlation between RBD‐specific IgM and IgG responses (Figure S4; Methods in the Appendix S1). While we could not find any correlation between age and S‐ and RBD‐specific IgM or IgG levels (Figure S5), it was interesting to note that RBD‐specific IgG and IgM levels were significantly correlated with the duration of COVID‐19 symptoms suggesting that prolonged disease and thus virus‐load may lead to increased virus‐specific antibody production (Figure S6).

In a subset of sera, we could analyse antibody reactivity to 25 synthetic overlapping 25‐30 amino acids long peptides spanning the complete receptor‐binding subunit S1, including RBD (Table S3, Figure S2 and Methods in the Appendix S1) indicating that there is no relevant peptide‐specific IgG or IgA reactivity detectable (Figures S7, S8 ). Sera from five tested convalescent COVID‐19 subjects and, to a lower degree, sera from subjects of control group P showed some IgM reactivity to peptides from the N‐ and C‐terminus of S1 and to distinct RBD‐derived peptides (Figures S7, S8). The amino acid sequences of the larger part of S1‐derived peptides from SARS‐CoV‐2 are highly conserved in SARS‐CoV but not in the other corona viruses known to cause common colds in humans (Figures [Link], [Link], [Link], [Link], [Link]) indicating, that the latter had not induced the peptide‐specific IgM responses. It is a limitation of our study that our ethics permission did not allow obtaining sputum or nasal secretion for the analysis of SARS‐CoV‐2‐specific secretory antibodies.

However, the interesting question for us was to study if and how many COVID‐19 convalescent patients develop antibodies which can inhibit the binding of the virus via RBD to the corresponding receptor ACE2 which would protect them from a recurrent infection. Since there is currently no accepted/standard virus neutralization assay authorized (FDA, July 3, 2020: https://www.cdc.gov/coronavirus/2019‐ncov/lab/resources/antibody‐tests‐guidelines.html), we developed a molecular interaction assay mimicking SARS‐CoV‐2 binding to its receptor ACE2 to investigate if COVID‐19 convalescent patients develop antibodies that can inhibit the binding of the virus‐derived receptor‐binding domain (RBD) to its receptor ACE2. This ELISA assay is based on plate‐bound recombinant ACE2 which is allowed to bind to recombinant His‐tagged RBD (Figure 2A). Bound RBD is then detected with a mouse monoclonal anti‐His antibody followed by a secondary HRP‐labelled anti‐mouse IgG_1_ antibody (Figure 2A and methods in this article´s Online Repository). This assay is similar to an interaction assay which recently became available (https://www.creative‐diagnostics.com/sars‐cov‐2‐inhibitor‐screening‐eia‐kit‐278105‐466.htm; https://www.researchsquare.com/article/rs‐24574/v1).

**FIGURE 2:**
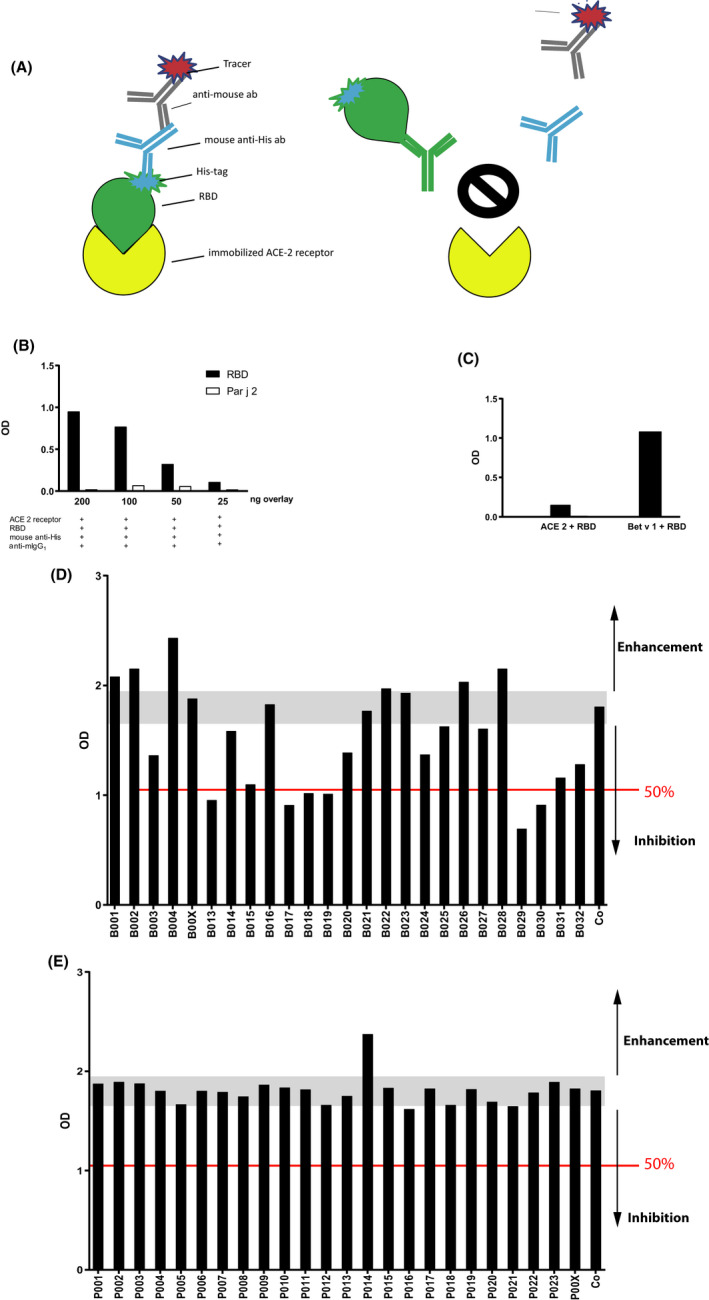
Molecular interaction assay based on ACE2 and SARS‐CoV‐2 RBD. (A) Scheme of the molecular interaction assay. ELISA plate‐bound recombinant ACE2 is incubated with His‐tagged recombinant SARS‐CoV‐2 RBD which is detected with a mouse monoclonal anti‐His‐tag antibody followed by HRP‐labelled anti‐mouse antibodies. (B) Specific binding of three different concentrations of RBD vs a control protein (Par j 2) (y‐axis: OD values correspond to bound RBD) to ACE2. Reactants and concentrations in ng/ml are summarized below the x‐axis. (C) Inhibition of RBD binding (y‐axis: OD values) to plate‐bound ACE2 by soluble ACE2 (ACE2 + RBD) vs a control protein (Bet v 1 + RBD). (D) Effects of serum antibodies from COVID‐19 convalescent subjects (group B) and (E) from subjects obtained before the COVID pandemic (group P, historic controls) on the ACE2‐RBD interaction. Shown is the binding of RBD to ACE2 (y‐axis: OD values correspond to amounts of ACE2‐bound RBD) which had been pre‐incubated with sera or buffer without serum (Co) (x‐axis). Each result is an average of duplicate determinations with <5% difference between the two values. The grey bar indicates the area of no alteration of RBD binding to ACE2 including the 10% variability of the assay. The arrows pointing downwards from the grey bar indicate the extent of inhibition and the red line marks 50% inhibition of RBD binding to ACE2. The arrows point upwards of the grey bars show enhancement of RBD binding to ACE2

Figure 2B shows that RBD binds to ACE2 in a dose‐dependent and specific manner whereas a negative control protein, the cysteine‐containing, His‐tagged recombinant *Parietaria* allergen, Par j 2, did not bind to ACE2 (Methods, Appendix S1). Next, we investigated whether binding of RBD to ACE2 can be blocked specifically by pre‐incubation with soluble ACE2 (Figure 2C and Methods in the Appendix S1). We found that pre‐incubation of RBD with ACE2 almost completely inhibited RBD binding to plate‐bound ACE2 whereas pre‐incubation with a negative control protein, recombinant major birch pollen allergen, Bet v 1, did not affect binding of RBD to ACE2 (Figure 2C).

We then studied the effects of antibodies in serum samples of COVID‐19 convalescent patients on the binding of RBD to ACE2. Figure 2D and Table S2 show the optical density (OD) values corresponding to the binding of RBD after pre‐incubation with sera from the 25 COVID‐19 convalescent patients to ACE2. A more than 50% inhibition was found for six sera (B013, B017, B018, B019, B029, B030), an up to 50% inhibition was found for nine sera (B003, B014, B015, B020, B024, B025, B027, B031, B032), no inhibition was found for five sera (B00X, B016, B021, B022, B023) and for five sera (B001, B002, B004, B026, B028) we noted even an enhancement of RBD binding to ACE2 (Figure 2D, Table S2). No relevant inhibition was observed for the 24 historic control sera obtained before the COVID‐19 pandemic indicating a high specificity of our assay (100%) (Figure 2E).

One serum (ie P0014) from the control group which contained elevated S‐ and RBD‐specific IgM antibodies caused an enhancement of RBD binding to ACE2 (Figure 2E, Table S2) pointing to the existence of “immune‐enhancing” natural anti‐glycan antibodies. Interestingly, neither the levels of S‐ nor RBD‐specific IgG or IgM antibodies were correlated with the inhibition of the binding of RBD to ACE2 in the inhibition assay (Figure S14). There were also no significant correlations between the percentages of inhibition of RBD binding to ACE2 and the duration of COVID‐19 symptoms and the age of the subjects, respectively (Figure S15).

There is a need for assays that can inform about characteristics of SARS‐CoV‐2‐specific antibodies such as possible protective effects and to detect potentially immune‐enhancing antibodies. The assay developed by us like another recently described similar assay (https://www.creative‐diagnostics.com/sars‐cov‐2‐inhibitor‐screening‐eia‐kit‐278105‐466.htm; https://www.researchsquare.com/article/rs‐24574/v1) would be simple and robust ELISA‐based molecular interaction assays which may allow testing for antibodies and compounds capable of inhibiting the binding of SARS‐CoV‐2 RBD to its receptor. This is important because certain molecules such as ACE2 derivatives or recombinant antibodies are being considered for treatment of COVID‐19 infections and there is a need to identify more and distil out the most efficient compounds for treatment.^4^, ^5^ Once these tests can be validated they may be also useful to characterize and identify COVID‐19 convalescent subjects producing antibodies capable of inhibiting the virus‐receptor interaction for obtaining therapeutic convalescent plasma and validating polyclonal immunoglobulin and monoclonal antibody preparations. Furthermore, once validated these assays could be suitable for a mass screening of COVID‐19 convalescent subjects regarding the presence of antibodies which prevent binding of the spike protein to the ACE2 receptor considering the possibility of future outbreaks of the virus. Our data, although limited, would rather indicate that the natural SARS‐CoV‐2 infection does not establish an antibody response in all infected subjects which can prevent the receptor‐virus interaction. Only 60% of COVID‐19‐convalescent patients had produced antibodies that inhibited the binding of RBD to ACE2. Since we could not perform additional virus neutralization tests, we cannot exclude the possibility that COVID‐19 convalescent subjects produce other types of protective antibodies besides those inhibiting RBD‐ACE2 binding. For example, there may be antibodies that may inhibit the fusion of the virus with the cell membrane or such contributing to virus clearance via Fc‐receptors. However, our study is the first to provide evidence for an increase in RBD binding to ACE2 caused by sera from patients who produced RBD‐specific IgG antibodies. This could be explained by the formation of immune complexes consisting of RBD and antibodies that bind to RBD without blocking the receptor interaction and eventually may be directed to carbohydrate epitopes of the virus. Such a mechanism of immune complex‐enhanced SARS‐CoV‐2 receptor binding would explain earlier findings of immune enhancement in COVID‐19.^6^ It is also conceivable that such an immune complex‐mediated cross‐linking of infected cells or cells containing ACE2‐bound virus could be responsible for the inexplicably high incidence of thromboembolic events as observed in patients suffering from severe COVID‐19 despite massive anticoagulation.^7^ In this context, it should be mentioned that ACE2 is expressed on vascular endothelial cells.^8^ However, studies are needed to investigate whether antibody‐mediated increases of RBD binding to ACE2 have a clinical relevance.

In summary, our findings suggest that a natural SARS‐CoV‐2 infection, similar to that observed previously for rhinovirus (RV) infections,^9^ does not induce a protective antibody response inhibiting the virus‐receptor interaction in all infected patients and therefore underline the urgent need for the development of a SARS‐CoV‐2 vaccine. The molecular interaction assays could be useful for identifying subjects having developed protective antibodies and for screening candidate vaccines to induce antibodies that inhibit the RBD‐ACE2 interaction once they have been validated.

## CONFLICT OF INTEREST

Rudolf Valenta has received research grants from HVD Life‐Sciences, Vienna, Austria, and from Viravaxx, Vienna, Austria. He serves as consultant for Viravaxx. Rainer Henning and Renata Kiss are employees of Viravaxx, Vienna, Austria. The other authors have no conflict of interest to declare.

## Supporting information

App S1

Fig S1

Fig S2

Fig S3

Fig S4

Fig S5

Fig S6

Fig S7

Fig S8

Fig S9

Fig S10

Fig S11

Fig S12

Fig S13

Fig S14

Fig S15

Tab S1

Tab S2

Tab S3
