# Supplementary material for: Antibodies in serum of convalescent patients following mild COVID‐19 do not always prevent virus‐receptor binding
Source: Allergy. 2020 Aug 27;76(3):878–83. doi: 10.1111/all.14523 (PMC7984338; doi:10.1111/all.14523)
Supplement: Supplementary file 1 — App S1 [file ALL-76-878-s018.docx]

**Appendix S1**

**Online Repository**

**Antibodies in serum of convalescent patients following mild COVID-19 do not always prevent virus receptor binding**

Pia Gattinger, PhD^a^, Kristina Borochova MSc^a^, Yulia Dorofeeva, MD^a^, Rainer Henning, PhD^b^, Renata Kiss, MSc^b^, Bernhard Kratzer, PhD^c^, Bernhard Mühl, MD^d^, Thomas Perkmann, MD^e^, Doris Trapin, MSc^c^, Martina Trella^e^, Paul Ettel, Inna Tulaeva, MD^a, f^, Winfried F. Pickl, MD^c^, Rudolf Valenta, MD^a,f, g, h^,^*^

^a^Department of Pathophysiology and Allergy Research, Division of Immunopathology, Center for Pathophysiology, Infectiology and Immunology, Medical University of Vienna, Vienna, Austria

^b^Viravaxx, Vienna, Austria.

^c^Institute of Immunology, Center for Pathophysiology, Infectiology and Immunology, Medical University of Vienna, Vienna, Austria

^d^Labors.at, Vienna, Austria

^e^Department of Laboratory Medicine, Medical University of Vienna, Vienna,

Austria

^f^Laboratory for Immunopathology, Department of Clinical Immunology and Allergy, Sechenov First Moscow State Medical University, Moscow, Russia.

^g^NRC Institute of Immunology FMBA of Russia, Moscow, Russia;

^h^Karl Landsteiner University of Health Sciences, Krems, Austria.

* Corresponding author

E-mail: rudolf.valenta@meduniwien.ac.at

**Figure and Table legends**

**Table S1.** Demographic and clinical characterization of subjects

**FIG S1.** IgA reactivity (y-axes: OD values correspond to bound immunoglobulins) to S and RBD determined for COVID-19 convalescent patients (group B: B001-B00X) and for individuals from the historic control group P (P001, P002, P003, P00X).

**FIG S2.** Amino acid sequence and scheme of (**A**) the complete S protein and (**B**) the S1 protein. The hydrophobic leader peptide is indicated in yellow, S1 peptides are underlined and numbered according to Table E2 and the RBD is printed in green. Glycosylation sites are printed in bold.

**Table S2.** SARS-CoV-2-specific antibodies and their effects on the RBD-ACE2 interaction

**FIG S3.** Correlations between SARS-CoV-2 specific antibodies measured with Siemens, Atellica IM SARS-CoV-2 Total (COV2T) test (y-axes: relative light units = RLU) and (**A**) RBD-specific IgG antibody levels (x-axis: optical density OD levels) (left) or (**B**) RBD-specific IgM-antibody levels (x-axis: optical density levels) in COVID-19 convalescent subjects. r and p levels are indicated.

**Table S3.** Characterization of SARS-CoV-2-derived synthetic peptides. Peptides are numbered, amino acid sequences, length of the peptides, molecular weights and isoelectric points (pI) are indicated.

**FIG S4.** Correlations between S-specific IgM- (y-axis: optical density levels) and IgG antibody levels (x-axes: optical density OD levels) (left) and RBD-specific IgM- (y-axis: optical density levels) and IgG antibody levels (x-axes: optical density OD levels) (right) in COVID-19 convalescent subjects. r and p levels are indicated.

**FIG S5.** Correlations between S- and RBD-specific antibody levels and age of COVID-19-convalescent subjects. Shown are the correlations between age (x-axes) and S-specific IgG levels (y-axis, OD levels) (left upper part), S-specific IgM levels (left lower part), RBD-specific IgG levels (right upper part) and RBD-specific IgM levels (right lower part). r and p levels are indicated.

**FIG S6.** Correlations between (**A**) RBD-specific IgG antibody levels (y-axis) and duration of symptoms (x-axis: days) and (**B**) between RBD-specific IgM antibody levels (y-axis) and duration of symptoms (x-axis: days). r and p levels are indicated.

**FIG S7.** IgG, IgM and IgA reactivity (y-axes: OD values correspond to bound antibodies) to S, RBD and spike protein-derived synthetic peptides (1-25) (x-axes) determined for COVID-19 convalescent subjects (group B: B001, B002, B003, B00X, different colors).

**FIG S8.** IgG, IgM and IgA reactivity (y-axes: OD values correspond to bound antibodies) to S, RBD and spike protein-derived synthetic peptides (1-25) (x-axes) determined for sera from subjects obtained before the COVID-19 pandemic (group P: P001, P002, P003, P00X, different colors).

**FIG S9.** Alignment of the amino acid sequence of the S protein from SARS-CoV-2 with that of SARS-CoV. Identical amino acids are indicated by asterisks, dashes indicate gaps. Synthetic overlapping peptides spanning S1 are boxed and numbered (1-25) and the RBD is indicated in green letters.

**FIG S10.** Alignment of the amino acid sequence of the S protein from SARS-CoV-2 with that of HCoV-OC43. Identical amino acids are indicated by asterisks, dashes indicate gaps. Synthetic overlapping peptides spanning S1 are boxed and numbered (1-25) and the RBD is indicated in green letters.

**FIG S11.** Alignment of the amino acid sequence of the S protein from SARS-CoV-2 with that of HCoV-NL63. Identical amino acids are indicated by asterisks, dashes indicate gaps. Synthetic overlapping peptides spanning S1 are boxed and numbered (1-25) and the RBD is indicated in green letters.

**FIG S12.** Alignment of the amino acid sequence of the S protein from SARS-CoV-2 with that of HCoV-HKU1. Identical amino acids are indicated by asterisks, dashes indicate gaps. Synthetic overlapping peptides spanning S1 are boxed and numbered (1-25) and the RBD is indicated in green letters.

**FIG S13.** Alignment of the amino acid sequence of the S protein from SARS-CoV-2 with that of HCo-229-E. Identical amino acids are indicated by asterisks, dashes indicate gaps. Synthetic overlapping peptides spanning S1 are boxed and numbered (1-25) and the RBD is indicated in green letters.

**FIG S14.** Correlations between S- and RBD-specific IgG antibody levels (y-axes: optical density OD levels) and percentages of inhibition of RBD binding to ACE2 (x-axes) (upper panels) and S- and RBD-specific IgM (y-axes: OD levels) and percentages of inhibition of RBD binding to ACE2 (x-axes) (lower panels) in COVID-convalescent subjects. r and p levels are indicated.

**FIG S15.** Correlations between percentages of inhibition of RBD binding to ACE2 (y-axes) and duration of symptoms (x-axis) (left panel) and age (x-axis) (right panel) in COVID-convalescent subjects. r and p levels are indicated.

**Materials and Methods**

**Recombinant proteins and peptides**

Recombinant insect cell-expressed SARS-CoV-2 spike protein (S1+S2, His tagged) was purchased from Sino Biological Inc., Beijing, P.R.China. SARS-CoV-2 spike protein receptor binding domain (RBD, His tagged) and human ACE-2 Receptor (Fc-tagged), both expressed in human cells, were purchased from GenScript, New Jersey, U.S.

Overlapping 25-35mer peptides covering the amino acid sequence of SARS-CoV-2 spike protein S1 domain were produced by solid-phase-synthesis as previously described ^1^. Recombinant His-tagged *Parietaria* allergen Par j 2 was expressed and purified as described^2^ and served as a control protein for RBD because it contains also cysteine residues. Recombinant major birch pollen allergen, Bet v 1 was purchased from Biomay, Vienna, Austria and served as control protein for ACE2 because it also assumes a mixed alpha helical and beta sheet structure.

**Sera from COVID-19 convalescent subjects and control subjects**

Sera from COVID-19 convalescent subjects were obtained approximately 10 weeks after confirmation of a SARS-CoV-2 infection by PCR when the subjects had recovered completely (Table S1, group B: n=25). The study was approved by the Ethics committee of the Medical University of Vienna, Austria (EK 1302/2020) and signed informed consent was obtained from the study subjects.

Anonymized sera from subjects which had been obtained before the COVID-19 pandemic (i.e., historic control samples) were from the serum bank of the Division of Immunopathology(Table S1, group P: n=24), Department of Pathophysiology and Allergy Research, Medical University of Vienna which had been collected with permission of the Ethics committee of the Medical University of Vienna (EK1641/2014).

**Direct ELISA and automated measurement of SARS-CoV-2-specific antibodies**

Immunoglobulin response to SARS-CoV-2 S protein and peptides were measured by ELISA. S protein (Sino Biological), RBD (GenScript) and peptides (2 µg/ml) in bicarbonate buffer were coated overnight onto NUNC Maxisorb 96 well plates (Thermofisher, Thermo-Fisher Scientific, Waltham, MA, USA). Plates were washed 3 times with washing buffer (PBS, 0.05% Tween 20) and subsequently blocked for 2 hours with blocking buffer (PBS, 0.05% Tween 20, 3% BSA). Serum samples 1:20 diluted were applied and incubated for 2 hours.

To determine human IgG reactivity, plates were washed 3 times and incubated with 1: 1000 diluted HRP-conjugated anti-human IgG (BD, San Jose, CA, USA) for 2 hours. After washing 3 times, the plates were developed with ABTS (Sigma-Aldrich, St. Louis, MO, USA) optical density was measured at 405/492 nm with Infinite F50 ELISA reader (Tecan, Männedorf, Switzerland).

For detection of human IgM and IgA, the plates were washed 3 times after incubation with serum samples (1:20) and incubated over night with either 1: 1000 diluted purified mouse anti-human IgM (BD) or 1:1000 diluted purified mouse anti- human IgA (BD). After washing three times, plates were incubated for 2 hours with 1:1000 diluted HRP-linked anti mouse IgG_1_ antibody (GE Healthcare, Marlborough, MA, USA), developed with ABTS and optical density was measured. Measurements were performed in the increasing linear area of the curve. The mean optical density (O.D.) values corresponding to the amount of bound immunoglobulins were measured as described above. For each protein or peptide the corresponding buffer control plus three times standard deviation of duplicate determinations was subtracted as background threshold. All determinations were performed in duplicates and each result~~s~~ is an average of duplicate determinations with <5% difference between the two values.

In addition, SARS-CoV-2-specific antibodies were determined in serum samples with the fully automated Siemens, Atellica IM SARS-CoV-2 Total (COV2T) test as recommended by the manufacturer. There are currently several commercial assays for measuring SARS-CoV-2-specific antibodies available (https://www.cdc.gov/coronavirus/2019-ncov/cases-updates/commercial-lab

-surveys.html; <https://wwwnc.cdc.gov/eid/article/26/10/20-2211-f1>)^3, 4^. Among these types of assays the ADVIA Centaur COV2T assay from Siemens is a fully automated 1‑step, highly sensitive and specific antigen sandwich immunoassay (https://www.siemens-healthineers.com/laboratory-diagnostics/assays-by-diseases-conditions/infectious-disease-assays/cov2t-assay) using acridinium ester chemiluminescent technology, in which antigens are bridged by antibodies present in the patient sample. The solid phase contains a preformed complex of streptavidin-coated microparticles and biotinylated SARS-CoV-2 recombinant antigens. This reagent is used to capture anti-SARS-CoV-2 antibodies in the patient sample. The lite reagent contains acridinium-ester-labeled SARS-CoV-2 recombinant antigens used to detect anti-SARSCoV-2 antibodies bound to the solid phase. A direct relationship exists between the amount of SARS-CoV-2 antibodies present in the patient sample and the amount of relative light units (RLUs) detected by the system.

**Interaction assay**

ACE- 2 receptor (GenScript) was coated (2 µg/ml) in bicarbonate buffer overnight onto NUNC Maxisorb 96 well plates (Thermofisher). Plates were washed 3 times with washing buffer and subsequently blocked for 3 hours at RT with blocking buffer. Meanwhile serum samples were diluted 1:2 in PBS, 0.05% Tween 20, 1% BSA and incubated for 2 hours with 100 ng His-tagged RBD (GenScript). For control purposes, 10 µg/ml ACE 2 receptor (positive control) and 10 µg/ml Bet v 1 (negative control) were pre-incubated with 100 ng His-tagged RBD.

The overlay was performed by adding the pre-incubated RBD samples to the coated and blocked ACE- 2 receptor followed by incubation for 3 hours. The plates were washed and incubated overnight with 1:1000 diluted mouse anti-His tag antibody (Dianova, Hamburg, Germany). After 3 washing three times, 1:1000 diluted HRP-linked anti-mouse IgG_1_ antibody (GE Healthcare) was incubated 2 hours and detected by ABTS. The mean optical density (O.D.) values corresponding to the amount of bound RBD were measured at 405 nm and 492 nm (reference) in a TECAN Infinite F5 ELISA reader with the integrated software i-control 2.0 (Tecan Group Ltd., Männedorf, Switzerland). ACE 2 receptor and Bet v 1 served as positive and negative controls in the blocking experiments, respectively. From each measurement the buffer control (overlay without RBD) was subtracted. All determinations were performed in duplicates and results are shown as mean values with a variation of <5%. The percentages of inhibition were calculated as follows:

Percentage inhibition (%) = (OD_Bet v 1_- OD_Serum_)/(OD_Bet v 1_-OD_ACE2_)x100

**Statistical evaluation**

Correlations between specific antibody levels, percentages of inhibition and duration of symptoms were assessed by Spearman’s correlation coefficient (r) using GraphPad Prism software, version 7.00 for Windows, GraphPad Software, La Jolla, Calif . P values < 0.05 were considered as significant.

**Sequence alignment**

Sequence alignment of SARS-CoV-2 spike protein (Genbank accession: QHD43416.1) with SARS-CoV spike protein (UniProtKB accession: P59594.1) and spike proteins from human coronavirus strains hCoV -NL63 (Genbank accession: AAS58177.1), hCoV-OC43 (Genbank accession: AAR01015.1), hCoV-229E (Genbank accession: AAG48592.1) and huCoV-HKU1(NCBI Reference Sequence: YP_173238.1) was obtained with the multiple sequence alignment program T-coffee^5^ and adapted manually from ExPASy tool Boxshade. N-glycosylation sites in S1, RBD and S which are represented by the following amino acid motifs N-X-S/T, where X is any amino acid except P were highlighted in the sequences and the analysis of the SARS-CoV-2 spike has been recently reported^6^.

**References**

1. Gallerano D, Ndlovu P, Makupe I, Focke-Tejkl M, Fauland K, Wollmann E, et al. Comparison of the specificities of IgG, IgG-subclass, IgA and IgM reactivities in African and European HIV-infected individuals with an HIV-1 clade C proteome-based array. *PloS one* 2015; 10, e0117204.

2. Dorofeeva Y, Colombo P, Blanca M, Mari A, Khanferyan R, Valenta R et al. Expression and characterization of recombinant Par j 1 and Par j 2 resembling the allergenic epitopes of Parietaria judaica pollen. *Sci Rep.* 2019; 9:15043.

3. Meyer B, Torriani G, Yerly S, Mazza L, Calame A, Arm-Vernez I, et al. Validation of a commercially available SARS-CoV-2 serological immunoassay. *Clin Microbiol Infect.* 2020 Jun 27;S1198-743X(20)30368-2. doi: 10.1016/j.cmi.2020.06.024. Online ahead of print.

4. Zhang Z-L, Hou L-L, Li D-T, Li F-Z, et al. Diagnostic efficacy of anti-SARS-CoV-2 IgG/IgM test for COVID-19: A meta-analysis. *J Med Virol.* 2020 Jun 22;10.1002/jmv.26211. doi: 10.1002/jmv.26211. Online ahead of print.

5. [Notredame C](https://www.ncbi.nlm.nih.gov/pubmed/?term=Notredame%20C%5BAuthor%5D&cauthor=true&cauthor_uid=10964570), [Higgins DG](https://www.ncbi.nlm.nih.gov/pubmed/?term=Higgins%20DG%5BAuthor%5D&cauthor=true&cauthor_uid=10964570), [Heringa J](https://www.ncbi.nlm.nih.gov/pubmed/?term=Heringa%20J%5BAuthor%5D&cauthor=true&cauthor_uid=10964570). T-Coffee: A novel method for fast and accurate multiple sequence alignment. [*J Mol Biol.*](https://www.ncbi.nlm.nih.gov/pubmed/10964570) 2000; 302:205-17.

6. Watanabe Y, Allen JD, Wrapp D, McLellan JS, Crispin M. Site-specific glycan analysis of the SARS-CoV-2 spike. *Science.* 2020; May 4:eabb9983. doi: 10.1126/science.abb9983. Online ahead of print. PMID: 32366695
