# Supplementary figures and images for: Antibodies in serum of convalescent patients following mild COVID‐19 do not always prevent virus‐receptor binding
Source: Allergy. 2020 Aug 27;76(3):878–83. doi: 10.1111/all.14523 (PMC7984338; doi:10.1111/all.14523)

FIGURE S1.

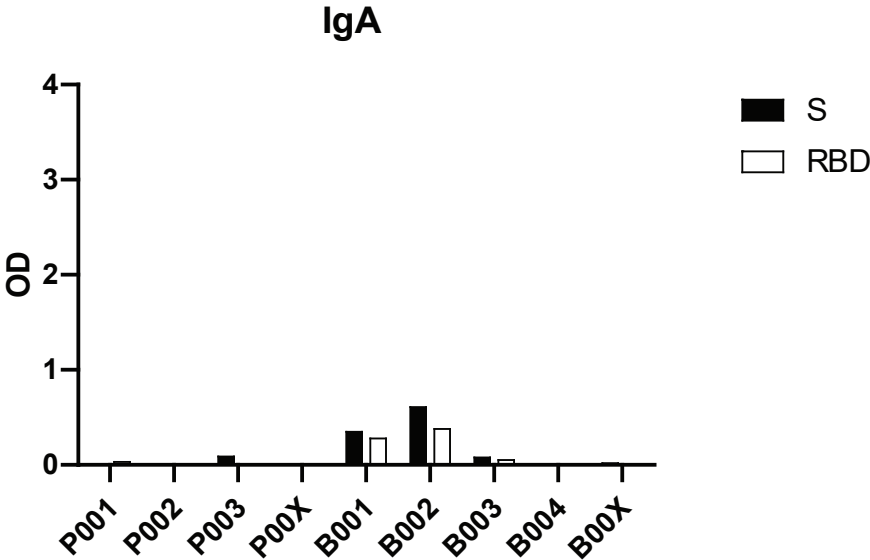

Supplement: Supplementary file 2 — Fig S1 [file ALL-76-878-s001.pdf]

FIGURE S3.

A

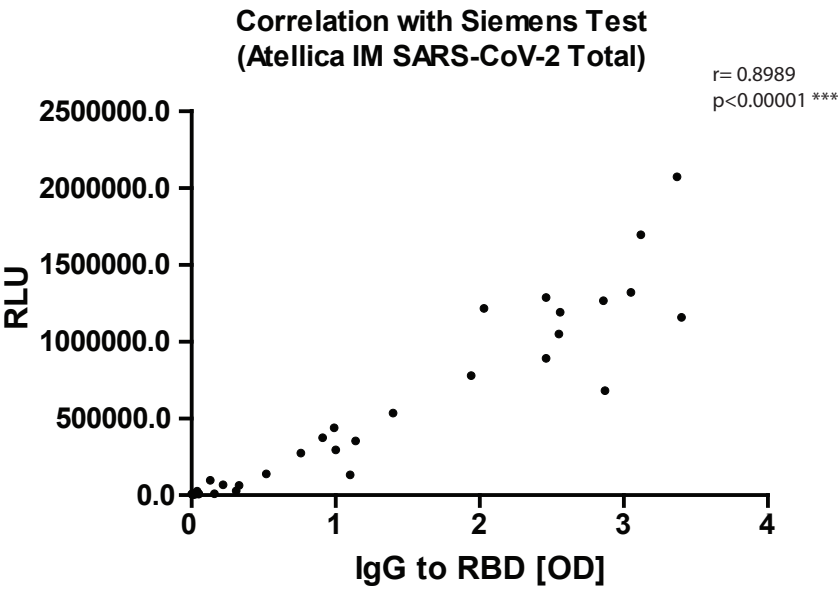

B

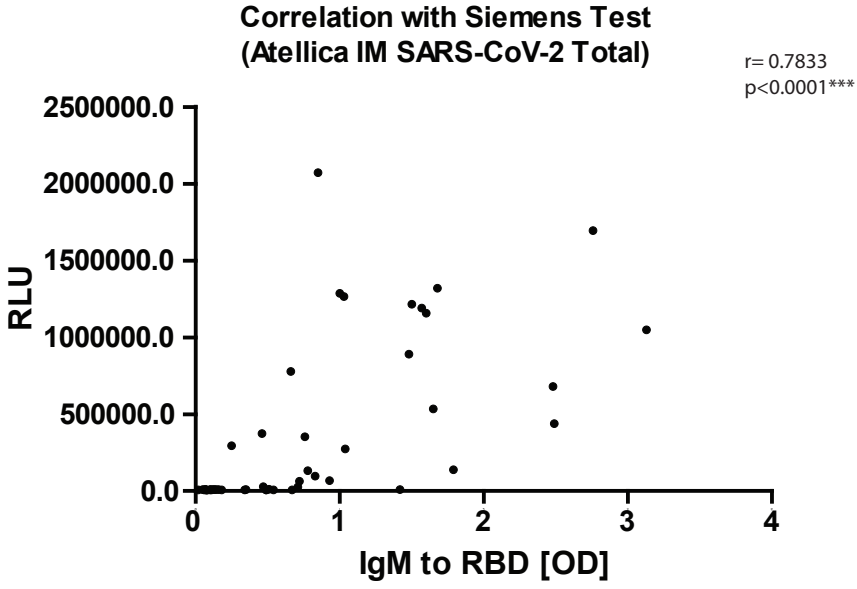

Supplement: Supplementary file 4 — Fig S3 [file ALL-76-878-s003.pdf]

FIGURE S4.

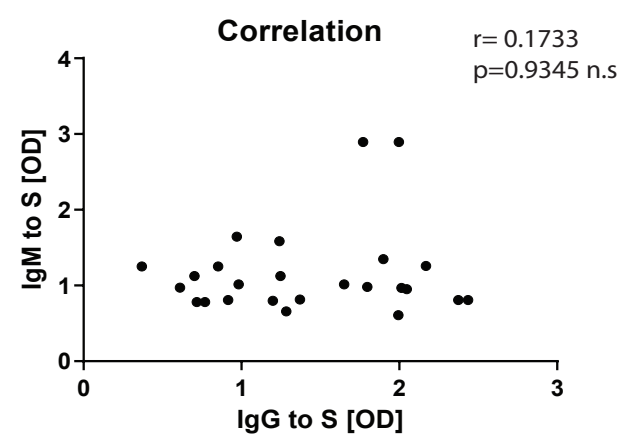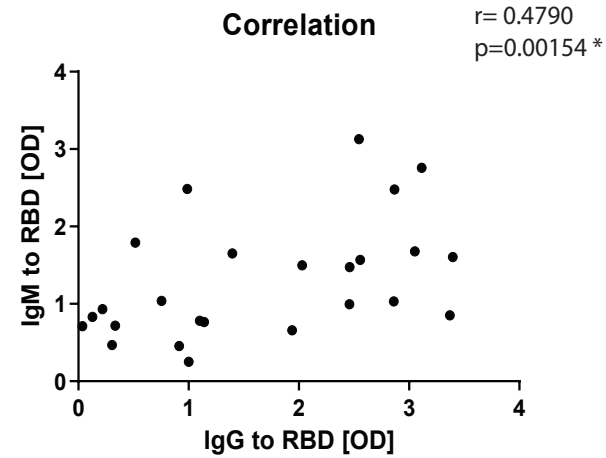

Supplement: Supplementary file 5 — Fig S4 [file ALL-76-878-s004.pdf]

FIGURE S5.

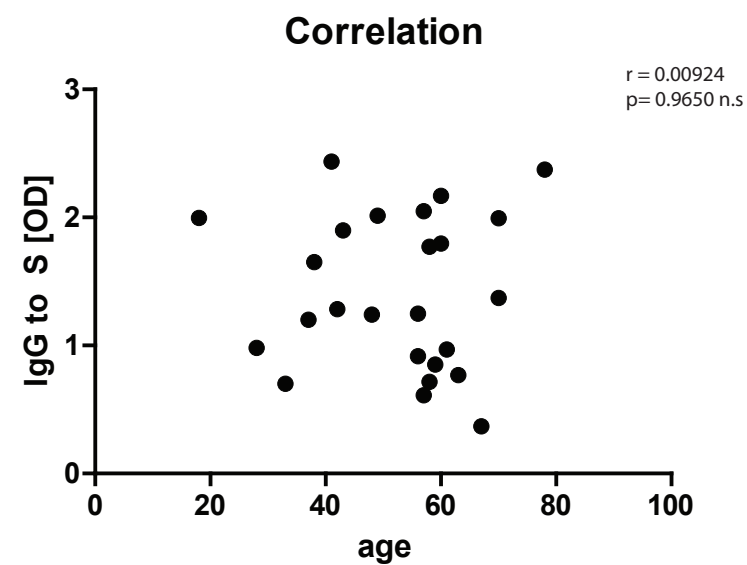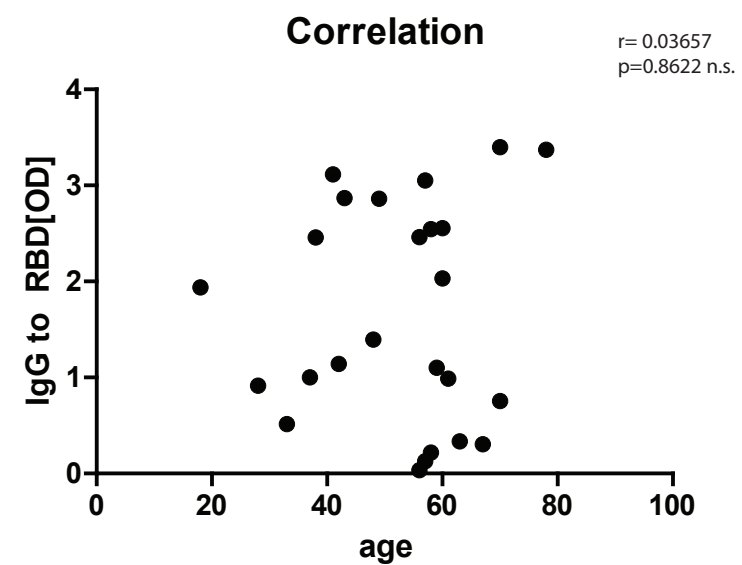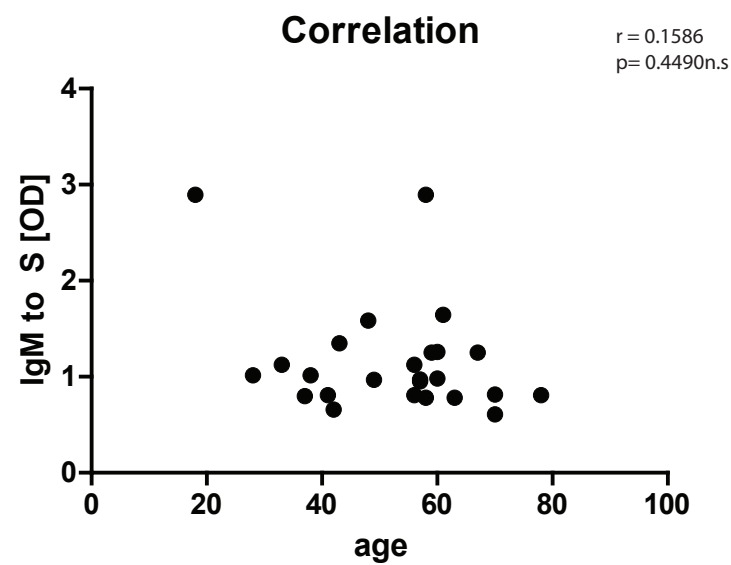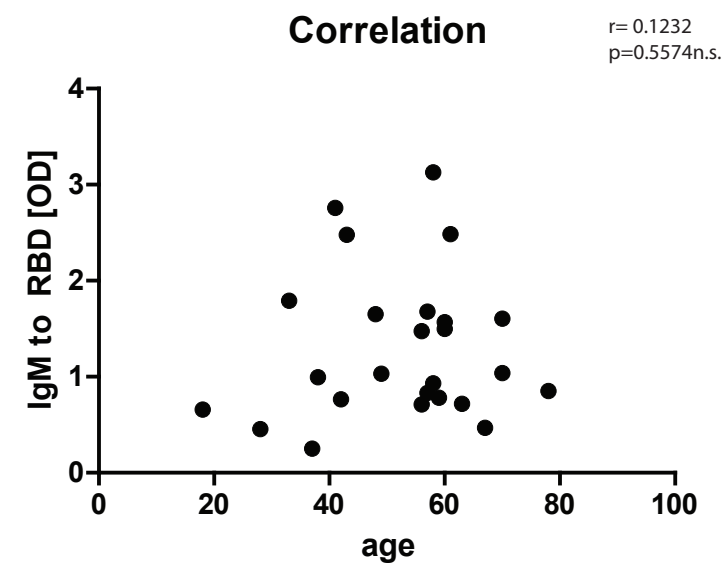

Supplement: Supplementary file 6 — Fig S5 [file ALL-76-878-s005.pdf]

FIGURE S6.

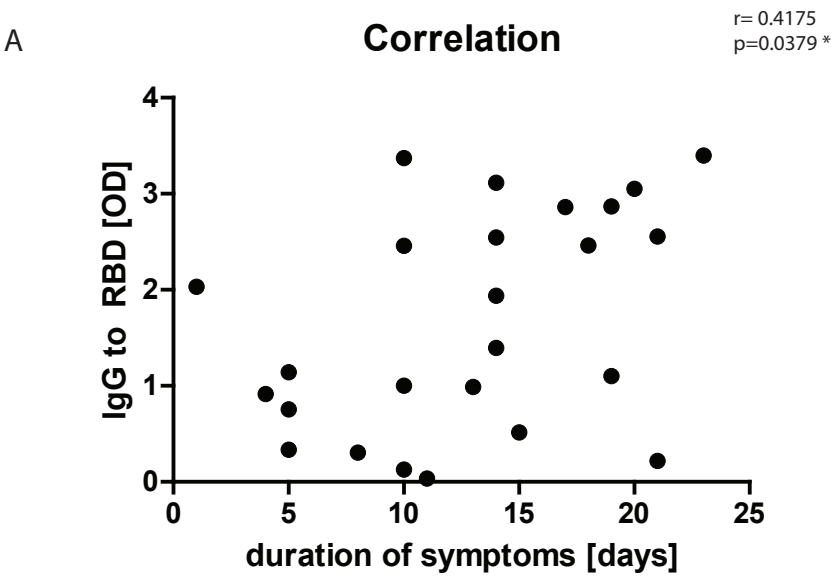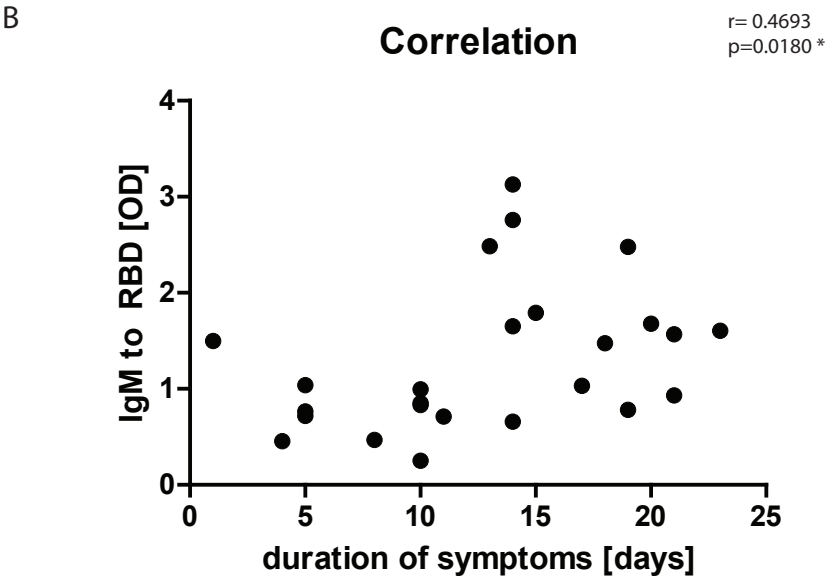

Supplement: Supplementary file 7 — Fig S6 [file ALL-76-878-s006.pdf]

FIGURE S7.

IgG

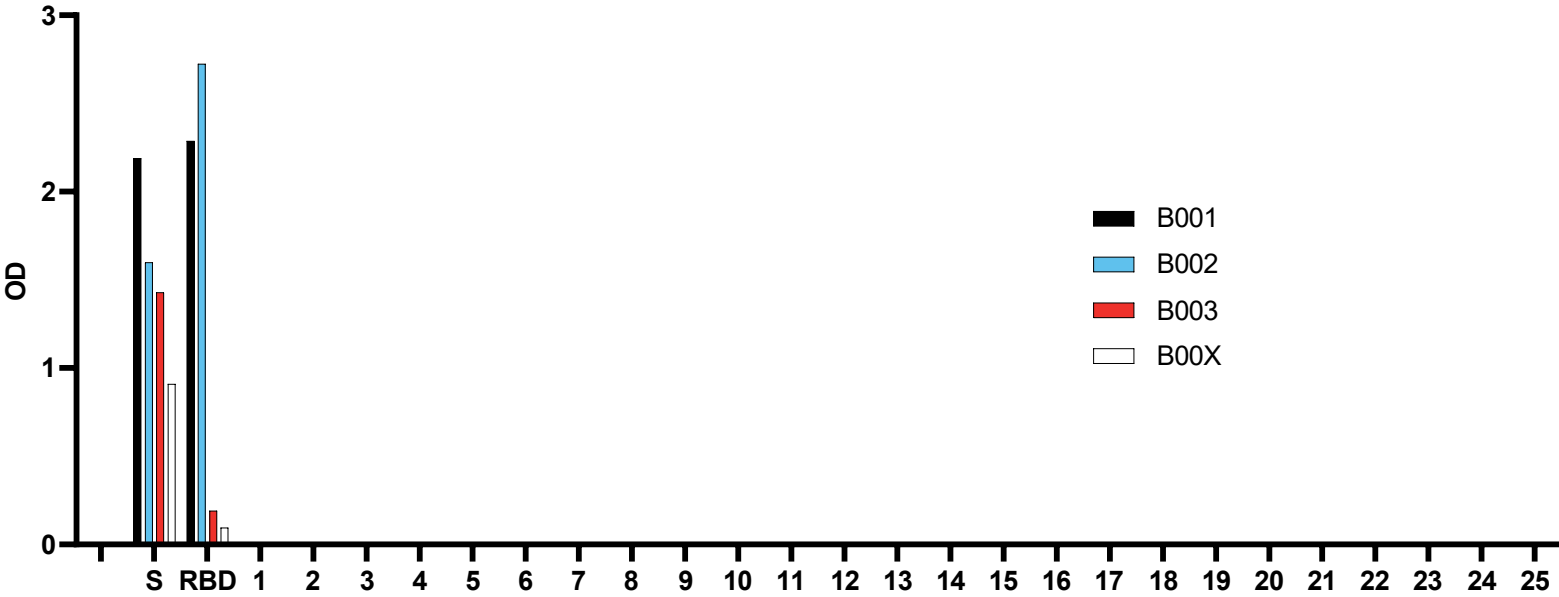

IgM

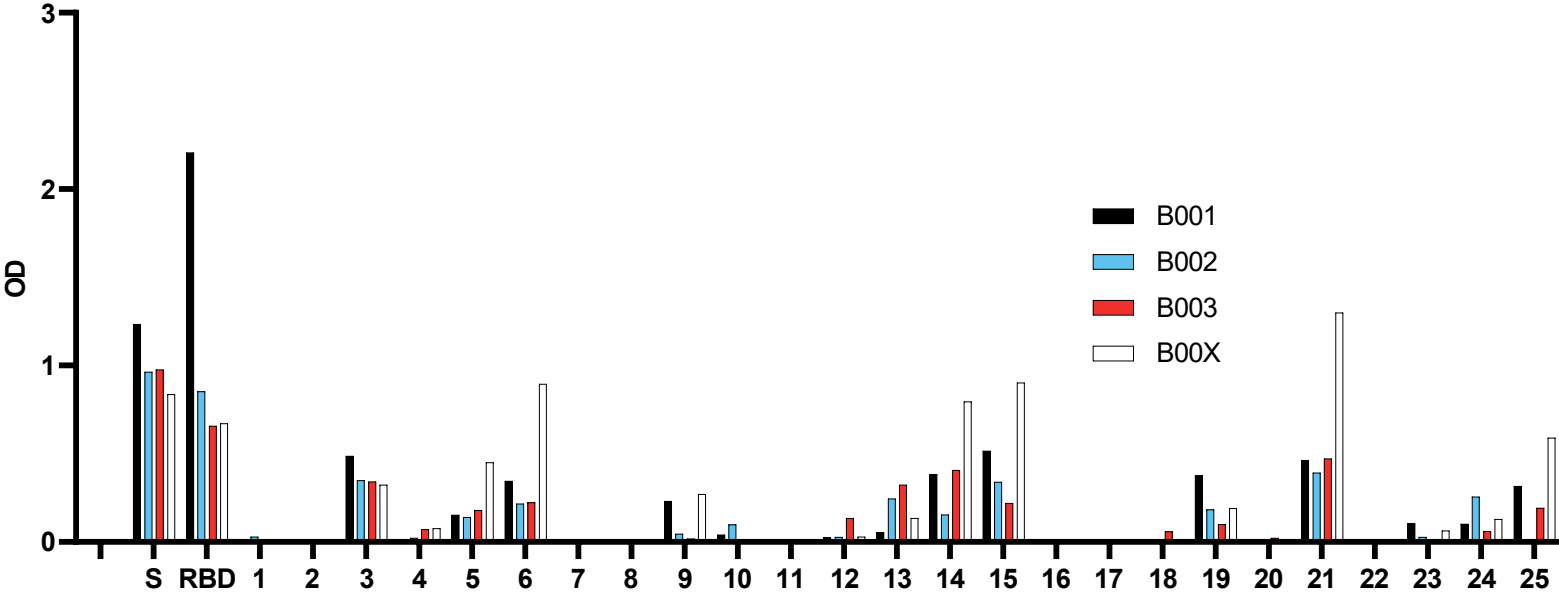

IgA

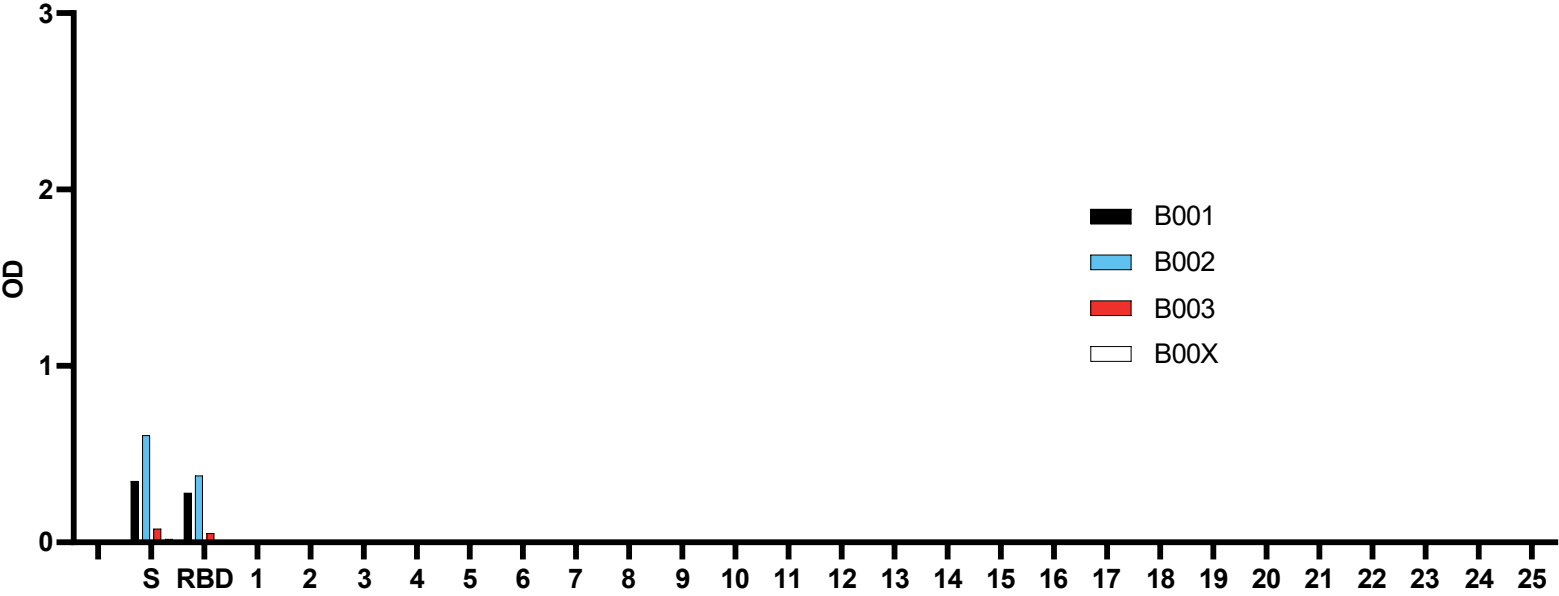

Supplement: Supplementary file 8 — Fig S7 [file ALL-76-878-s007.pdf]

FIGURE S8.

IgG

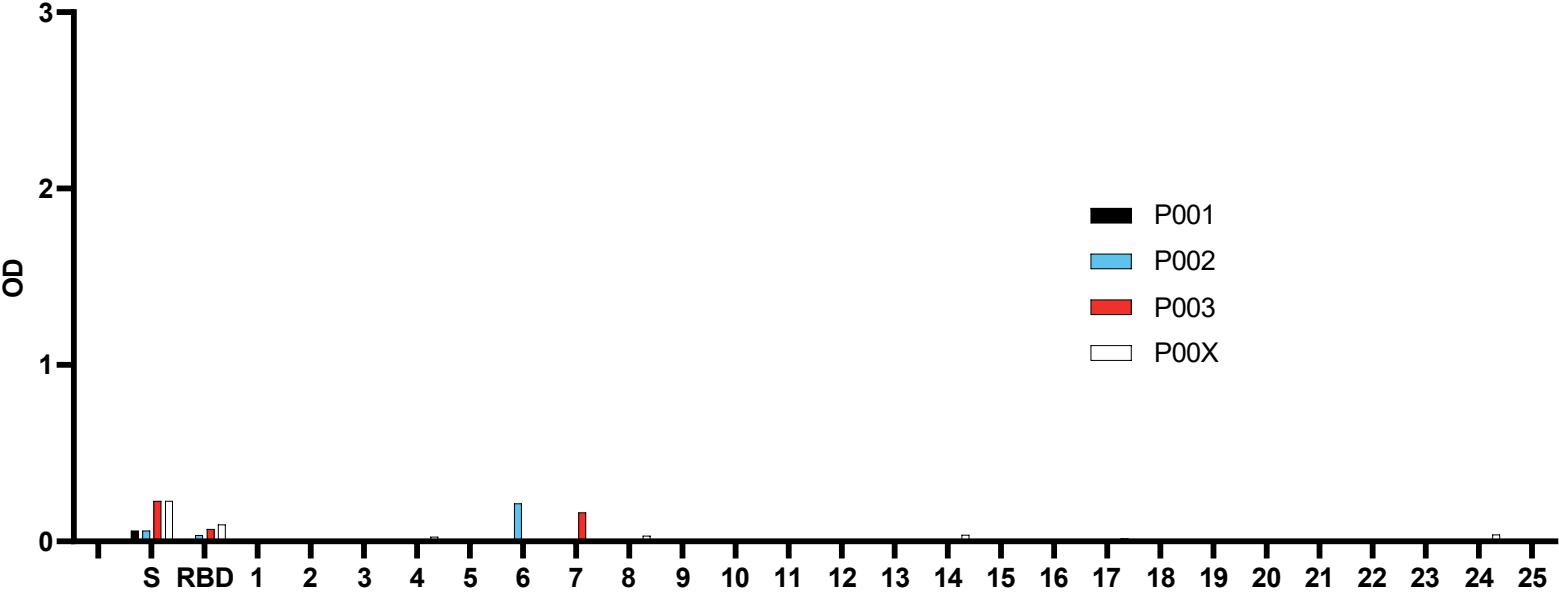

IgM

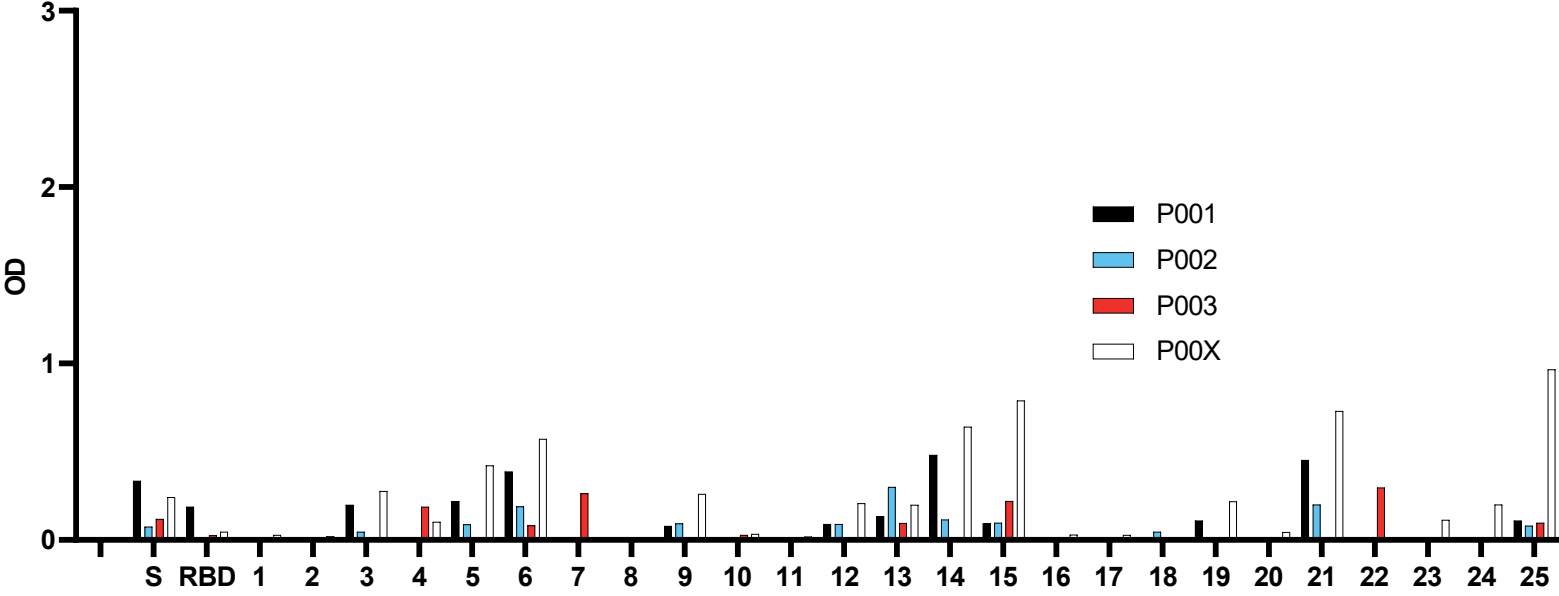

IgA

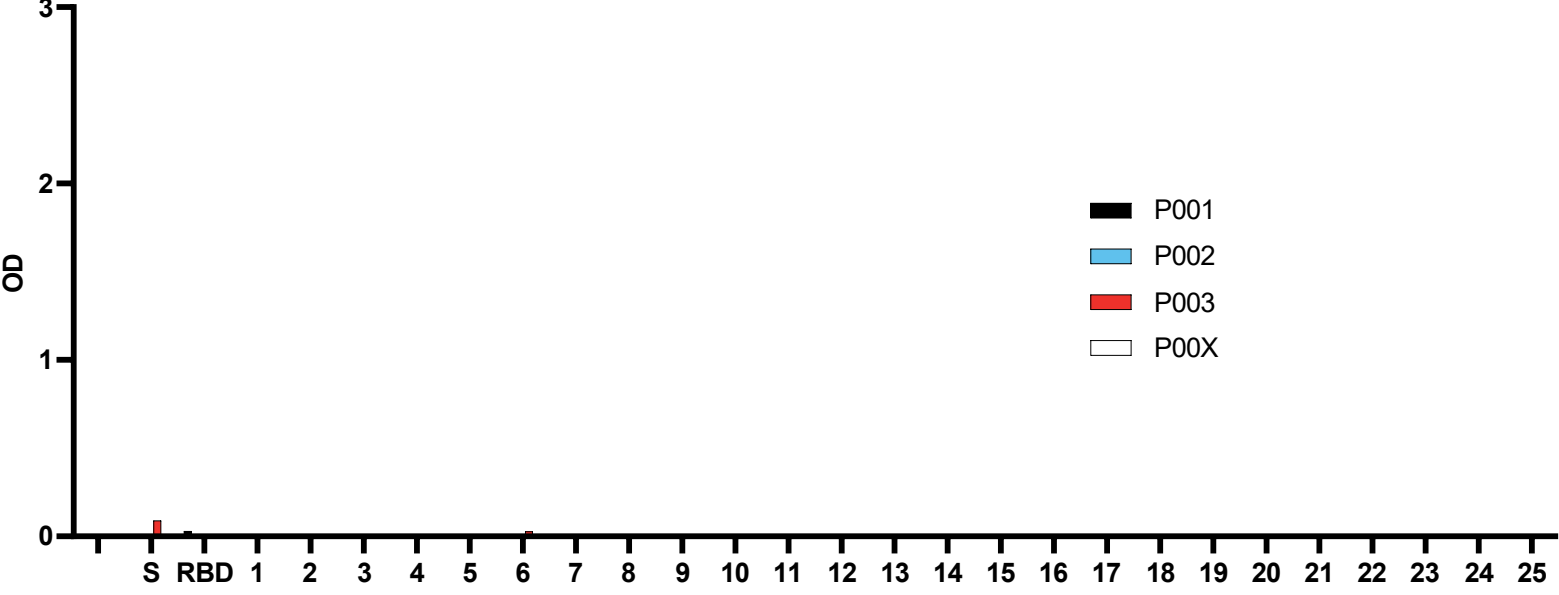

Supplement: Supplementary file 9 — Fig S8 [file ALL-76-878-s008.pdf]

FIG S14.

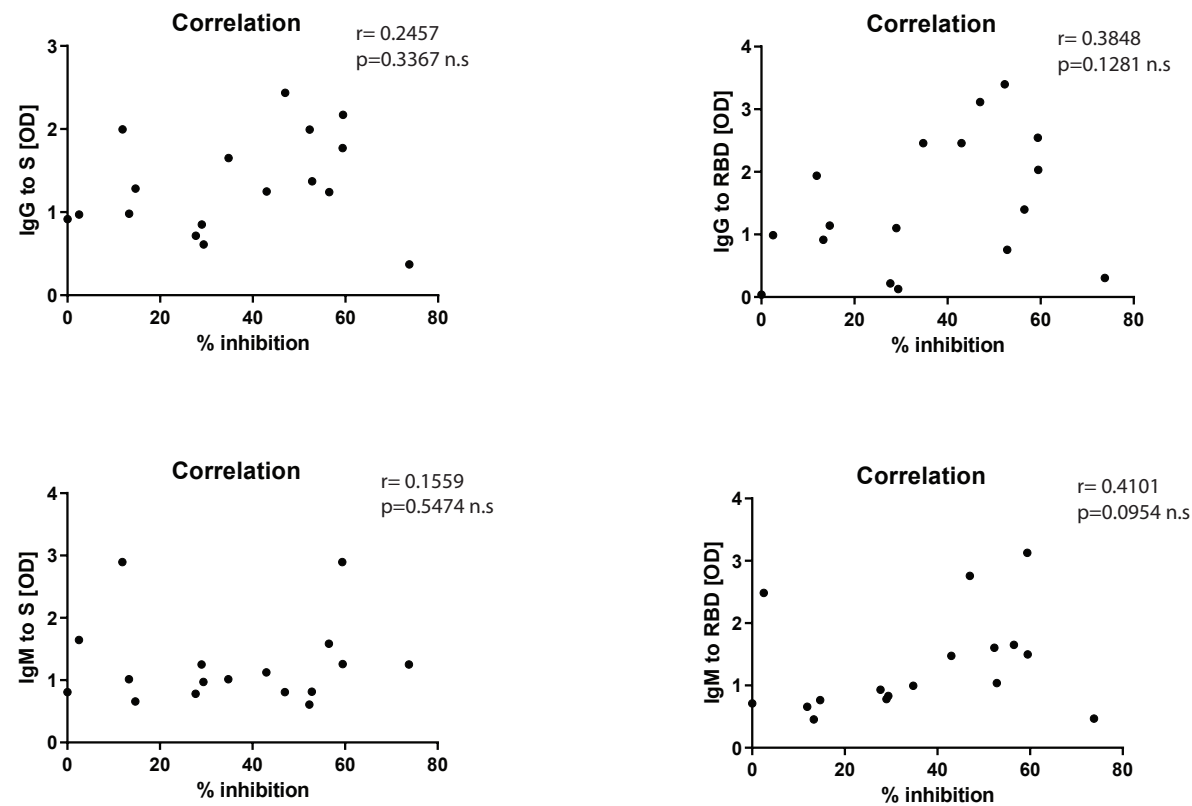

Supplement: Supplementary file 15 — Fig S14 [file ALL-76-878-s014.pdf]

FIGURE S15.

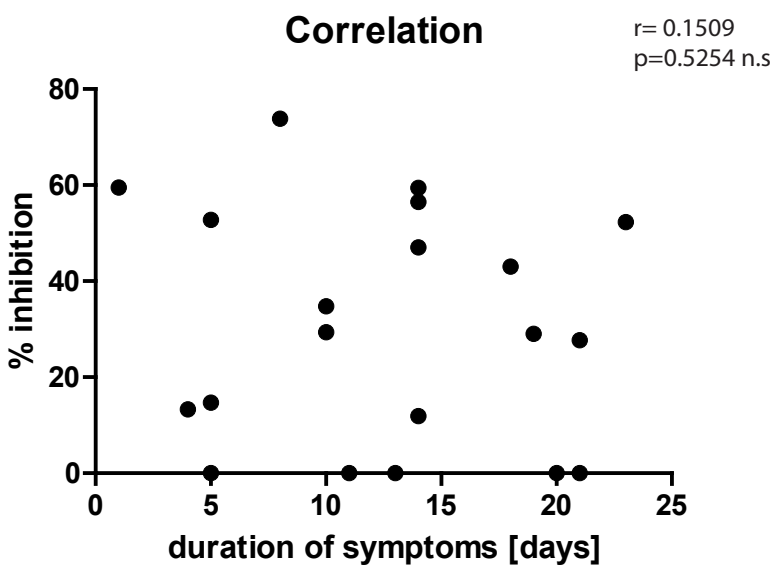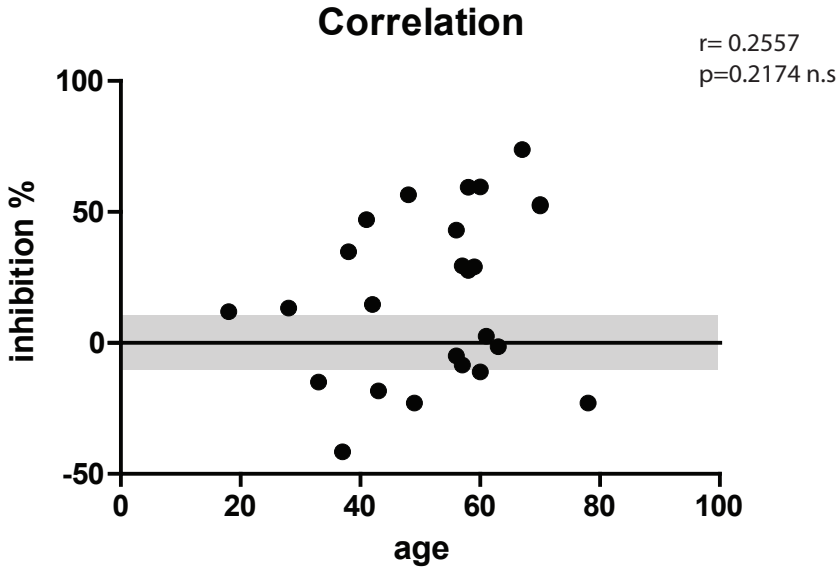

Supplement: Supplementary file 16 — Fig S15 [file ALL-76-878-s015.pdf]
