# Supplementary material for: Antibodies in serum of convalescent patients following mild COVID‐19 do not always prevent virus‐receptor binding
Source: Allergy. 2020 Aug 27;76(3):878–83. doi: 10.1111/all.14523 (PMC7984338; doi:10.1111/all.14523)
Supplement: Supplementary file 3 — Fig S2 [file ALL-76-878-s019.pdf]

FIGURE S2.

A

MFVFLVLLPLVSSQCV**NLT**TRTQLPPAYTNSFTRGVYYPDKVFRSSVLHSTQDLFL
PFFS**NVT**WFHAIHVSGT**NGT**KRFDNPVLPFNDGVYFASTEKSNIIRGWIFGTTLDS
KTQSLLIVN**NAT**NVVIKVCEFQFCNDPFLGVYYHKN**NKS**WMESEFRVYSSAN**NCT**F
EYVSQPFLMDLEGKQGNFKNLREFVFKNIDGYFKIYSKHTPINLVRDLPQGFSALE
PLVDLPIGI**NIT**RFQTLLALHRSYLTPGDSSSGWTAGAAAYYVGYLQPRTFLLKYN
E**NGT**ITDAVDCALDPLSETKCTLKSFTVEKGIYQTSNFRVQPTESIVRFP**NIT**NLC
PFGEVF**NAT**RFASVYAWNRKRISNCVADYSVLYNSASFSTFKCYGVSPTKLNDLCF
TNVYADSFVIRGDEVRQIAPGQTGKIADYNYKLPDDFTGCVIAWNSNNLDSKVGGN
YNYLYRLFRKSNLKPFERDISTEIYQAGSTPCNGVEGFNCYFPLQSYGFQPTNGVG
YQPYRVVVLSFELLHAPATVCGPKKSTNLVKNKCVNFNFNGLTGTGVLTESNKKFL
PFQQFGRDIADTTDAVRDPQTLEILDITPCSFGGVSVITPGT**NTS**NQVAVLYQDV**N**
CTEVPVAIHADQLTPTWRVYSTGSNVFQTRAGCLIGAEHV**NNS**YECDIPIGAGICA
SYQTQTNSPRRARSVASQSIIAYTMSLGAENSVAYS**NNS**IAIPT**NFT**ISVTTEILP
VSMTKTSVDCTMYICGDSTECSNLLLQYGSFCTQLNRALTGIAVEQDKNTQEVFAQ
VKQIYKTPPIKDFGGF**NFS**QILPDPSKPSKRSFIEDLLFNKVTLADAGFIKQYGDC
LGDIAARDLICAQKFNGLTVLPPLLTDEMIAQYTSALLAGTITSGWTFGAGAALQI
PFAMQMAYRFNGIGVTQNVLYENQKLIANQFNSAIGKIQDSLSSTASALGKLQDVV
NQNAQALNTLVKQLSSNFGAISSVLNDILSRLDKVEAEVQIDRLITGRLQSLQTYV
TQQLIRAAEIRASANLAATKMSECVLGQSKRVDFCGKGYHLMSFPQSAPHGVVFLH
VTYVPAQEK**NFT**TAPAICHDGKAHFPREGVFVS**NGT**HWFVTQRNFYEPQIITTDNT
FVSGNCDVVIGIV**NNT**VYDPLQPELDSFKEELDKYFK**NHT**SPDVDLGDISGI**NAS**V
VNIQKEIDRLNEVAKNL**NES**LIDLQELGKYEQYIKWPWYIWLGFIAGLIAIVMVTI
MLCCMTSCCSCLKGCCSCGSCCKFDEDDSEPVLKGVKLHYT

B

MFVFLVLLPLVSSQCV**NLT**TRTQLPPAYTNSFTRGVYYPDKVFRSSVLHSTQDLFLPFFS**NVT**WFHAIHV
1 2

SGT**NGT**KRFDNPVLPFNDGVYFASTEKSNIIRGWIFGTTLDSKTQSLLIVN**NAT**NVVIKVCEFQFCNDPF
3 4

LGVYYHKN**NKS**WMESEFRVYSSAN**NCT**FEYVSQPFLMDLEGKQGNFKNLREFVFKNIDGYFKIYSKHTPI
5 6 7

NLVRDLPQGFSALEPLVDLPIGI**NIT**RFQTLLALHRSYLTPGDSSSGWTAGAAAYYVGYLQPRTFLLKYN
8 9

E**NGT**ITDAVDCALDPLSETKCTLKSFTVEKGIY TSNFRVQPTESIVRFP**NIT**NLCPFGEVF**NAT**RFASV
10 11 12

YAWNRKRISNCVADYSVLYNSASFSTFKCYGVSPTKLNDLCFTNVYADSFVIRGDEVRQIAPGQTGKIAD
13 14 15

YNYKLPDDFTGCVIAWNSNNLDSKVGGNYNYLYRLFRKSNLKPFERDISTEIYQAGSTPCNGVEGFNCYF
16 18 17 19

PLQSYGFQPTNGVGYQPYRVVVLSFELLHAPATVCGPKKSTNLVKNKCVNFNFNGLTGTGVLTESNKKFL
20 21

PFQQFGRDIADTTDAVRDPQTLEILDITPCSFGGVSVITPGT**NTS**NQVAVLYQDV**NCT**EVPVAIHADQLT
22 23

PTWRVYSTGSNVFQTRAGCLIGAEHV**NNS**YECDIPIGAGICASYQTQTNSPRRARSVA
24 25
