# Supplementary material for: Antibodies in serum of convalescent patients following mild COVID‐19 do not always prevent virus‐receptor binding
Source: Allergy. 2020 Aug 27;76(3):878–83. doi: 10.1111/all.14523 (PMC7984338; doi:10.1111/all.14523)
Supplement: Supplementary file 10 — Fig S9 [file ALL-76-878-s009.pdf]

FIGUGE S9.

```
                                      1                                2
SARS_CoV_2      1 MFVFLVLLPLVSSQCVNLT-TRTQ-LPPAYT--NSFTRGVYYPDKVFRSSVLHSTQDLFL
SARS-CoV        1 **I**LF*T*T*GSDLDRCT*FDDVQA*N**QHT*SM*******EI***DT*YL******
                              3
SARS_CoV_2     57 PFFSNVTWFHAIHVSGTNGTKRFDNPVLPFNDGVYFASTEKSNIIRGWIFGTTLDSKTQS
SARS-CoV       61 **Y****G**T*------*H*--*G***I**K**I***A*****VV***V**S*MNN*SQ*
                             4                         5                 6
SARS_CoV_2    117 LLIVNNATNVVIKVCEFQFCNDPFLGVYYHKNNKSWMESEFRVYSSANNCTFEYVSQPFL
SARS-CoV      114 VI*INNS*****RA*N*EL*DN**FA*SK----PMGTQTHTMIFDN*F******ISDA*S
                                        7
SARS_CoV_2    177 MDLEGKQGNFKNLREFVFKNIDGYFKIYSKHTPINLVRDLPQGFSALEPLVDLPIGINIT
SARS-CoV      170 L*VSE*S****H********K**FLYV*KGYQ**DV*****S**NT*K*IFK**L*****
                               8                         9
SARS_CoV_2    237 RFQTLLALHRSYLTPGDSSSGWTAGAAAYYVGYLQPRTFLLKYNENGTITDAVDCALDPL
SARS-CoV      230 N*RAI*TAFS----*AQ--DI*GTS*****F****K*T**M***D*************SQN**
                          10                  11                12         13
SARS_CoV_2    297 SETKCTLKSFTVEKGIYQTSNFRVQPTESIVRFPNITNLCPFGEVFNATRFASVYAWNRK
SARS-CoV      284 A*L**SV***EID***********V*SGDV*******************K*P*****E**
                                     14                        15
SARS_CoV_2    357 RISNCVADYSVLYNSASFSTFKCYGVSPTKLNDLCFTNVYADSFVIRGDEVRQIAPGQTG
SARS-CoV      344 K**************TF************A********S********VK**DV**********
                    16                    17                   18               19
SARS_CoV_2    417 KIADYNYKLPDDFTGCVIAWNSNNLDSKVGGNYNYLYRLFRKSNLKPFERDISTEIYQAG
SARS-CoV      404 V***********M**VL***TR*I*ATST*****K**YL*HGK*R*******NVPFSPD
                                      20                          21
SARS_CoV_2    477 STPCNGVEGFNCYFPLQSYGFQPTNGVGYQPYRVVVLSFELLHAPATVCGPKKSTNLVKN
SARS-CoV      464 GK**TP-PAL***W**ND***YT*T*I*****************N*********L**D*I**
                                                        22
SARS_CoV_2    537 KCVNFNFNGLTGTGVLTESNKKFLPFQQFGRDIADTTDAVRDPQTLEILDITPCSFGGVS
SARS-CoV      523 Q***************P*S*R*Q********VS*F**S****K*S*****S********
                                              23                           24
SARS_CoV_2    597 VITPGTNTSNQVAVLYQDVNCTEVPVAIHADQLTPTWRVYSTGSNVFQTRAGCLIGAEHV
SARS-CoV      583 *******A*SE***********D*ST*********A**I*****N*****Q**********
                                    25
SARS_CoV_2    657 NNSYECDIPIGAGICASYQTQTNSPRRARSVASQSIIAYTMSLGAENSVAYSNNSIAIPT
SARS-CoV      643 DT****************H*VS----LL**TSQK**V********DS*I*****T*****

SARS_CoV_2    717 NFTISVTTEILPVSMTKTSVDCTMYICGDSTECSNLLLQYGSFCTQLNRALTGIAVEQDK
SARS-CoV      699 **S**I***VM****A******N**********A*****************S***A***R

SARS_CoV_2    777 NTQEVFAQVKQIYKTPPIKDFGGFNFSQILPDPSKPSKRSFIEDLLFNKVTLADAGFIKQ
SARS-CoV      759 **R********M****TL*Y*************L**T*********************M**

SARS_CoV_2    837 YGDCLGDIAARDLICAQKFNGLTVLPPLLTDEMIAQYTSALLAGTITSGWTFGAGAALQI
SARS-CoV      819 **E*****N***********************D***A**A**VS**A*A************

SARS_CoV_2    897 PFAMQMAYRFNGIGVTQNVLYENQKLIANQFNSAIGKIQDSLSSTASALGKLQDVVNQNA
SARS-CoV      879 ************************Q******K**SQ**E**TT*ST*************

SARS_CoV_2    957 QALNTLVKQLSSNFGAISSVLNDILSRLDKVEAEVQIDRLITGRLQSLQTYVTQQLIRAA
SARS-CoV      939 ************************************************************

SARS_CoV_2   1017 EIRASANLAATKMSECVLGQSKRVDFCGKGYHLMSFPQSAPHGVVFLHVTYVPAQEKNFT
SARS-CoV      999 ************************************A**************S**R***

SARS_CoV_2   1077 TAPAICHDGKAHFPREGVFVSNGTHWFVTQRNFYEPQIITTDNTFVSGNCDVVIGIVNNT
SARS-CoV     1059 *******E***Y*********F***S**I*****FS**********************I***

SARS_CoV_2   1137 VYDPLQPELDSFKEELDKYFKNHTSPDVDLGDISGINASVVNIQKEIDRLNEVAKNLNES
SARS-CoV     1119 ************************************************************

SARS_CoV_2   1197 LIDLQELGKYEQYIKWPWYIWLGFIAGLIAIVMVTIMLCCMTSCCSCLKGCCSCGSCCKF
SARS-CoV     1179 ******************V*****************L**************A*********

SARS_CoV_2   1257 DEDDSEPVLKGVKLHYT
SARS-CoV     1239 *****************
```
