# Supplementary material for: Antibodies in serum of convalescent patients following mild COVID‐19 do not always prevent virus‐receptor binding
Source: Allergy. 2020 Aug 27;76(3):878–83. doi: 10.1111/all.14523 (PMC7984338; doi:10.1111/all.14523)
Supplement: Supplementary file 11 — Fig S10 [file ALL-76-878-s010.pdf]

FIGURE S10.

```
                                        1
SARS_CoV_2    1 MFVFL-VLLPLVSSQCVNL----------TTRTQLPPAYTNSF-----------------
OC43          1 **LI*LIS**TAFAVIGD*KCTSDTSYINDKD*GP**IS*DTVDVTNGLGTYYVLDRVYL

                                            2                    3
SARS_CoV_2   33 -----------TRGVYYPDKVFRSSVLHSTQDLFLPFFSNVTWFHAIHVSGTNGTKRFDN
OC43         61 NTTLFLNGYYP*S*ST*RNMALKG***L*RLWFKP**L*DF-------------------

                                                                4
SARS_CoV_2   82 PVLPFNDGVYFASTEKSNIIRGWIFGTTLDSKTQSLLIVNNATNVVI-------------
OC43        102 -----IN*I-**KVKNTKV*KDRVMYSEFPAI*IGSTF**TSYS**VQPRTINSTQDGYN

                                         5                         6
SARS_CoV_2  129 --------KVCEFQFCNDPFLGVYYHKNNKSWMESEFRVYSSANNCTFEYVSQPFLMDLE
OC43        156 KLQGLLEVS**QYNM*EY*Q--TIC*P*LGNHRKELWHLDTGVVS*LYK---RN*TY*VN

                                    7
SARS_CoV_2  181 GKQGNFKNLREFVFKNIDGYFKIYSKHTPINLVRDLPQGFSALEPLVDLPIGINITRFQT
OC43        211 AD------YLY*H*YQEG*T*YA*FTD*GV--*T---------KF*FNVYL---------

                           8                        9                 10
SARS_CoV_2  241 LLALHRSYLTPGDSSSGWTAGA-AAYYVGYLQPRTFLLKYNENGTITDAVDCALDPLSET
OC43        245 GM**SHY*VM*LTCN*KVKN*FTLE*W*TP*TS*QY**AF*QD*I*FN****MS*FM**I

                                 11                    12                13
SARS_CoV_2  300 KCTLKSFTVEKGIYQTSNFRVQPTESIVRFPNITNLCPFGEVFNATRFASVYAWNRKRIS
OC43        305 **KTQ*IAPPT*V*ELNGYT***IADVY*RKLNLPN*NIEAWL*DKSVP*PLN*E**TF*

                               14                     15                 16
SARS_CoV_2  360 NCVADYSVLYNSASFSTFKCYGVSPTKLNDLCFTNVYADSFVIRGDEVRQIAPGQTGKIA
OC43        365 **NFNM*S*MSFIQADS*T*NNIDAA*IYGM**SSITI*K*A*PNGRKVDLQL*NL*YLQ

                                   17                 18                 19
SARS_CoV_2  420 DYNYKLPDDFTGCVIAWNSNNLDSKVG----GNYNYLYRLFRKSNLKPFERDISTEIYQA
OC43        425 SF**RIDTTA*S*QLYY*LPAANVS*SRFNPSTW*KRFGFIED*VF**RPAGVL*NHDVV

                                    20                                  21
SARS_CoV_2  476 GSTPCNGVEGFNCYFPLQ--S--YGFQPTNGVGYQPYRVVVLSFELLHAPATV-------
OC43        485 YAQH*FKAPKNF*PCK*NGSCVGS*PGKN**I*TC*AGTNY*TCDN*CT*DPITFKATGT

SARS_CoV_2  525 --------------------------------CGP-------------------------
OC43        545 YKCPQTKSLVGIGEHCSGLAVKSDYCGGNSCT*R*QAFLGWSADSCLQGDKCNIFANFIL

                                                                       22
SARS_CoV_2  528 --------------KKSTNLVKNKCVNFNFNGLTGTGVLTESNKKFL-PFQQFGRDIADT
OC43        605 HDVNSGLTCSTDLQ*AN*DIILGV***YDLY*IL*Q*IFV*V*ATYYNSW*NLLY*SNGN

                                                            23
SARS_CoV_2  573 TDAVRDPQTLEILDITPCSFGGVSVITPGTNTSNQVAVLYQDVNCTEVPVAIHADQLTPT
OC43        665 LYGF**YI*NRTFM*RS*YS*R**AAFHA--N*SEP*L*FRNIK*NY*FNNSLTR**Q*-

                                   24                         25
SARS_CoV_2  633 WRVYSTGSNVFQTRAGCLIGAEHVN--NSYECDIPIGAGICASYQTQTNSPRRARSVASQ
OC43        722 -------I*Y*DSYL**VVN*YNSTAISVQT**LTV*S*Y*VD*SKN----**S*GAITT

SARS_CoV_2  691 SIIAYTMSLGAENSVAYS------NNSIAIPTNFTISVTTEILPVSMTKTSVDCTMYICG
OC43        771 GYRFTNFEPFTV***ND*LEPVGGLYE*Q**SE***GNME*FIQT*SP*VTI**AAFV**

SARS_CoV_2  745 DSTECSNLLLQYGSFCTQLNRALTGIAVEQDKNTQEVF-AQVKQIYKTPPIKDFGGFNFS
OC43        831 *YAA*KSQ*VE*****DNI*AI**EVNELL*TTQLQ*ANSLMNGVTLSTKL**GVN**VD

SARS_CoV_2  804 QILP---------DPSKPSKRSFIEDLLFNKVTLADAGFIKQYGDCLGDIAARDLICAQK
OC43        891 D*NFSPVLGCLGSEC**A*S**A******D**K*S*V**VEA*NN*T*GAEI*****V*S

SARS_CoV_2  855 FNGLTVLPPLLTDEMIAQYTSALLAGTITSGWTFGAGAALQIPFAMQMAYRFNGIGVTQN
OC43        951 YK*IK******SENQ*SG**L*ATSASLFPL**AA----AGV**YLNVQ**I**L***MD

SARS_CoV_2  915 VLYENQKLIANQFNSAIGKIQDSLSSTASALGKLQDVVNQNAQALNTLVKQLSSNFGAIS
OC43       1007 **SQ*******A**N*LYA**EGFDA*N***V*I*A***A**E***N*LQ***NR*****

SARS_CoV_2  975 SVLNDILSRLDKVEAEVQIDRLITGRLQSLQTYVTQQLIRAAEIRASANLAATKMSECVL
OC43       1067 AS*QE******AL***A******N***TA*NA**S***SDSTLVKF**AQ*ME*VN***K

SARS_CoV_2 1035 GQSKRVDFCGKGYHLMSFPQSAPHGVVFLHVTYVPAQEKNFTTAPAICHDGK-AHFPREG
OC43       1127 S**S*IN***N*N*II*LV*N**Y*LY*I*FS***TKYVTARVS*GL*IA*DRGIA*KS*

SARS_CoV_2 1094 VFVSNGTHWFVTQRNFYEPQIITTDNTFVSGNCDVVIGIVNNTVYDPLQPELDSFKEELD
OC43       1187 Y**NVNNT*MY*GSGY*Y*EP**EN*VV*MST*A*NYTKAPYVMLNTSI*N*PD******

SARS_CoV_2 1154 KYFKNHTSPDVDLGDISGINASVVNIQKEIDRLNEVAKNLNESLIDLQELGKYEQYIKWP
OC43       1247 QW***Q**VAP**S-LDY**VTFLDL*V*MN**Q*AI*V**Q*Y*N*KDI*T**Y*V***

SARS_CoV_2 1214 WYIWLGFIAGLIAIVMVTIMLCCMTSC-CSCLKGCCSCGSCCKFDEDDSEPVLKGVKLHY
OC43       1306 **V**LICLAGV*MLVLLFFI**C*G*GT**F*K---**G**DDYTGYQ*L*I*TSHD--

SARS_CoV_2 1273 T
OC43       1361 D
```
