# Supplementary material for: Antibodies in serum of convalescent patients following mild COVID‐19 do not always prevent virus‐receptor binding
Source: Allergy. 2020 Aug 27;76(3):878–83. doi: 10.1111/all.14523 (PMC7984338; doi:10.1111/all.14523)
Supplement: Supplementary file 12 — Fig S11 [file ALL-76-878-s011.pdf]

FIGURE S11.

```
                                    1                              2
SARS_CoV_2    1  MFVFLVLLPLVSSQCV-------NLTTRTQLPPAYTNSFTRGVYYPDKVFR---SSVLHS
NL63          1  *KL**I**V*PLAS*FFTCNSNA**SMLQLGV*DNSSTIVT*L-L*THW*CANQ*TSVY*
                                                  3
SARS_CoV_2   51  TQDLFLPFFSNVTWFHAIH---------------VSGTNGTKRFD--------------N
NL63         60  ANGF*YIDVG*HRSAF*L*TGYYDANQYYIYVTNEI*L*ASVTLKICKFSRNTTFDFLS*
                                                            4
SARS_CoV_2   82  PVLPFNDGVYFASTEKSNIIRGWIFGTTLDSKTQSLLIVN--------------------
NL63        120  ASSS*DCI*NLLF**Q----L*APL*I*ISGE*VR*HLY*VTRTFYVPAAYKLTKLSVKC
                                                                 5
SARS_CoV_2  122  -----------------NATNVVIKVCEFQFCND---------------------PFLGV
NL63        176  YFNYSCVFSVVNATVTV*V*THNGR*VNYTV*D*CNGYTDNIFSVQQDGRIPNGF**NNW
                                             6
SARS_CoV_2  144  YYHKNNKSWMESEFRVYSSA--------------------------NNCT-F--EYVSQP
NL63        236  FLLT*GSTLVDGVS*L*QPLRLTCLWPVPGLKSSTGFVYFNATGSDV**NGYQHNS*VDV
                                            7
SARS_CoV_2  175  FLMDLEGKQGNFKNLRE--FVFKNIDGYFKIYSKHTPIN-LVRDLPQGFSALEPLVDLPI
NL63        296  MRYN*NFSANSLD**KSGVI***TLQYDVLF*CSNSSSGV*DTTI*F*P*SQPYYCFINS
                       8                                       9
SARS_CoV_2  232  GINITRFQTLLALHRSY---LTPGDSSSGWTAGAAAYYVGYLQPRTFLL-----------
NL63        356  T**T*HVS*FVGILPPTVREIVVARTGQFYIN*FKYFDL*FIEAVN*NVTTASATDFWTV
                                              10                         11
SARS_CoV_2  278  --------KYNENG-TITDAVDCALDPLSETKCTLKSFTVEKGIYQTSNFR---VQPTES
NL63        416  AFATFVDVLV*VSATN*QNLLY*-DS*FEKLQ*EHLQ*GLQD*F*S-A**LDDN*L*-*T
                           12               13                  14
SARS_CoV_2  326  IVRFPNITNLCPFGEVFNATRFASVYAWNRKRISNCVADYSVLYNSASFSTFKCYGVSPT
NL63        473  Y*AL*IYYQHTDINFT-------------------------AT***G-GS**VCK*H
                           15                            16          17
SARS_CoV_2  386  KLNDLCFT----NVYADSFVIRG--DEVRQIAPGQTGKIADYNYKLPDDFTGCVIAWNSN
NL63        504  QV*ISLNGNTSVC*RTSH*S**YIYNR*KSGS**DS------------------------
                    18                          19               20
SARS_CoV_2  440  NLDSKVGGNYNYLYRLFRKSNLKPFERDISTEIYQAGSTPCN-GVEG-FNCYFPLQSYGF
NL63        540  ------------SWHIYL**GTC**SFSKL-NNF*KFK*I*FST**VPGS*N***EATWH
                                             21
SARS_CoV_2  498  QPTNGVGYQPYRVVVLSFELLHAPATVCGPKKSTNLVKNKCVNFNFNGLTGTGVLTESNK
NL63        587  YTSYTIVGAL*VTWSEGNSITGV*YP*S*IREFS***L*N*TKY*IYDYV***IIRS**Q
                                      22
SARS_CoV_2  558  KFLPFQQFGRDIADTTDAVRDPQTLEILDITPCSFGGVSVITPGTNTSNQVAVLYQDVNC
NL63        647  SLAGGITYVS------------NSGNL*GFKNV*T*NIFIV**-C*QPD****YQ*SIIG
                              23                          24
SARS_CoV_2  618  TEVP-----VAIHADQLTPTWRVYSTGSNVFQTRAGCLIGAEHVNNSYECDIPIGAGICA
NL63        694  AMTAVNESRYGLQNLLQL*NFYYV*N*G*NCT*AVMT---------------YSNF****
                        25
SARS_CoV_2  673  SYQTQTNSPRRARSVASQSIIAYTMSLGAENSVAYSNNSIAIPTNFTISVTTEILPVSMT
NL63        739  DGSLIPVR**NSSDNGISA**T---------------ANLS**S*W*T**QV*Y*QITS*

SARS_CoV_2  733  KTSVDCTMYICGDSTECSNLLLQYGSFCTQLNRALTGIAVEQDKNTQEVFAQVKQIYKTP
NL63        784  PIV***AT*V*NGNPR*K***K**T*A*KTIED**RLS*HLETNDVSSMLTFDSNAFSLA

SARS_CoV_2  793  PIKDFGGFNFSQILPDP----SKPSKRSFIEDLLFNKVTLADAGFIK-QYGDCLGDIAAR
NL63        844  NVTS**DY*L*SV**QRNIRS*RIAG**AL*****S**VTSGL*TVDVD*KS*TKGLSIA

SARS_CoV_2  848  DLICAQKFNGLTVLPPLLTDEMIAQYTSALLAGTITSGWTFGAGAALQIPFAMQMAYRFN
NL63        904  **A***YY**IM***GVADA*RM*M**GS*IG*MVLG*L*S----*AA***SLALQA*L*

SARS_CoV_2  908  GIGVTQNVLYENQKLIANQFNSAIGKIQDSLSSTA--------------SALGKLQDVVN
NL63        960  YVALQTD**Q****IL*AS**K**NN*VA*FSSVNDAITQTAEAIHTVTI**N*I*****

SARS_CoV_2  954  QNAQALNTLVKQLSSNFGAISSVLNDILSRLDKVEAEVQIDRLITGRLQSLQTYVTQQLI
NL63       1020  *QGS***H*TS**RH**Q***NSIQA*YD***SIQ*DQ*V********AA*NAF*S*V*N

SARS_CoV_2 1014  RAAEIRASANLAATKMSECVLGQSKRVDFCGKGYHLMSFPQSAPHGVVFLHVTYVPAQEK
NL63       1080  KYT*V*G*RR**QQ*IN***KS**N*YG***N*T*IF*IVN***D*LL***TVLL*TDY*

SARS_CoV_2 1074  NFTTAPAICHDGKAHF-PRE--GVFVSNGTHWFVTQRNFYEPQIITTDNTFVSGNCDVVI
NL63       1140  *VKAWSG**V**IYGYVL*QPNL*LY*DNGVFR**S*VMFQ*RLPVLSDFVQIY**N*TF

SARS_CoV_2 1131  GIVNNTVYDPLQPE-------LDSFKEELDKYFKNHTSPDVDLGDISGINASVVNIQKEI
NL63       1200  VNISRVELHTVI*DYVDVNKT*QE*AQN*P**V----K*NF**---TPF*LTYL*LSS*L

SARS_CoV_2 1184  DRLNE--------------VAKNLNESLIDLQELGKYEQYIKWPWYIWLGFIAGLIAIVM
NL63       1253  KQ*EAKTASLFQTTVELQGLIDQI*STYV**KL*NRF*N******WV**I-*SVVFVVLL

SARS_CoV_2 1230  VTIMLCCM-TSCCSC-------LKGCCSCGSCCKFDEDDSEPVLKGVKLHYT
NL63       1312  SLLVF**LS*G**G*CNCLTSSMR***D***T-*L------*YYEFE*V*VQ
```
