# Supplementary material for: Antibodies in serum of convalescent patients following mild COVID‐19 do not always prevent virus‐receptor binding
Source: Allergy. 2020 Aug 27;76(3):878–83. doi: 10.1111/all.14523 (PMC7984338; doi:10.1111/all.14523)
Supplement: Supplementary file 13 — Fig S12 [file ALL-76-878-s012.pdf]

FIGURE S12.

```
SARS_CoV_2      1 MFVFLVLLPLVS-----SQCVNLTT---RTQLP--PAY--TNSFTRGVYYP-DKVFRSSV
HKU1            1 *LLIIFI**TTLAVIGDFN*T*FAINDLN*TV*RISE*VVDV*YGL*T**IL*R*YLN--

SARS_CoV_2     48 LHSTQDLFLPFFSN-VTWFHAIHVSGTN--GTKRFDNPVLP-FNDGVYFAS---------
HKU1           59 ---*TI**TGY*PKSGAN*RDLSLK**TYLS*LWYQK*F*SD**N*IFSRVKNTKLYVNK

SARS_CoV_2     95 ---TEKSNIIRGWIFGTTLDSKTQSLLIVNNATNVVIKVCEFQFCNDPFLGV--YYHKNN
HKU1          116 TLYS*F*T*VI*SV*----INNSYTIVVQPHNGVLE*TA*QYTM*EY*HTICKSKGSSR*

SARS_CoV_2    150 KSWMESEFR-VYSSANNCTFEYVSQPFLMDLEGKQGNFKNLREFVFKNIDGYFKIYSKH-
HKU1          172 E**HFDKSEPLCLFKK*F*YNVS-T-------------DWLY*H*YQER*T*YA*YADS

SARS_CoV_2    208 -TPINLVRDLPQGFSALEPLVDLPIGINITRFQTLLALHRSYLTPGDSSSGWTAGAAAYY
HKU1          217 GM*TTFLFS-------------*YL*TLLSHYYV------LP**CNAI**NTDNETLQ*W

SARS_CoV_2    267 VGYLQPRTFLLKYNENGTITDAVDCALDPLSETKCTLKSFTVEKGIYQTSNFRVQPTESI
HKU1          258 *TP*SK*QY***FDNR*V**N*****SSSFF**IQ*KT**LLPNT*V*DL*G*T*K*VATV

SARS_CoV_2    327 V-RFPNITNLCPFGEVFNATRFASVYAWNRKRISNCVADYSVLYNSASFSTFKCYGVSPT
HKU1          318 HR*I*DLPD-*DIDKWL*NFNVP*PLN*E**IF***NFNL*T*LRLVHTDS*S*NNFDES

SARS_CoV_2    386 KLNDLCFTNVYADSFVIRGDEVRQIAPGQTGKIADYNYKLPDDFTGCVIAWNSNNLDSKV
HKU1          377 *IYGS**KSIVL*K*A*PNSRRSDLQL*SS*FLQSS***IDTTSSS*QLYYSLPAINVTI

SARS_CoV_2    446 GGNYNYLYRLFRKSNLKPFERDISTEIYQAGSTPCNGVEGFNCYFPLQS-----------
HKU1          437 -N***PS-SWN*RYGFNN*NLSSHSVV*S---RY*FS*NNTF*--*CAKPSFASSCKSHK

SARS_CoV_2    495 --------------------------------------------YGFQPTNGVGYQ----
HKU1          490 PPSASCPIGTNYRSCESTTVLDHTDWCRCSCLPDPITAYDPRSCSQKKSLV***EHCAGF

SARS_CoV_2    507 -----------------------------------PYRVVVLSFELLHA--PATVCGP--
HKU1          550 GVDEEKCGVLDGSYNVSCLCSTDAFLGWSYDTCVSNN*CNIF*NFI*NG**SG*T*SNDL

SARS_CoV_2    528 -KKSTNLVKNKCVNFNFNGLTGTGVLTESNKK-FLPFQQFGRDIADTTDAVRDPQTLEIL
HKU1          610 LQPN*EVFTDV**DYDLY*I**Q*IFK*VSAVYYNSW*NLLY*SNGNIIGFK*FV*NKTY

SARS_CoV_2    586 DITPCSFGGVSVITPGTNTSNQVAVLYQDVNCTEVPVAIHADQLTPTWRVYSTGSNVFQT
HKU1          670 N*F**YA*R**AAF--HQNASSL*L**RNLK*SY*LNN*S-----------L*TQPY*DS

SARS_CoV_2    646 RAGCLIGAEHVNNS--YECDIPIGAGICASYQTQTN-SPRRARSVASQSIIAYTMSLGAE
HKU1          717 YL**VFN*DNLTDYSVSS*ALRM*S*F*VD*NSPSSS*S**K*RSI*A*Y---RFVTFEP

SARS_CoV_2    703 NSVAYSNN---------SIAIPTNFTISVTTEILPVSMTKTSVDCTMYICGDSTECSNLL
HKU1          774 FN*SFV*DSIESVGGLYE*K*******VGQE*FIQTNSP*VTI**SLFV*SNYAA*HD**

SARS_CoV_2    754 LQYGSFCTQLNRALTGIAVEQDKNTQEVFAQVK------QIYKTPPIKDFGGFNFSQIL-
HKU1          834 SE**T**DNI*SI*DEVNGLL*TTQLH*ADTLMQGVTLSSNLN*NLHF*VDNI**KSLVG

SARS_CoV_2    807 --PDPSKPSKRSFIEDLLFNKVTLADAGFIKQYGDCLGDIAARDLICAQKFNGLTVLPPL
HKU1          894 CLGPHCGS*S***F*****D**K*S*V**VEA*NN*T*GSEI***L*V*S***IK****I

SARS_CoV_2    865 LTDEMIAQYTSALLAGTITSGWTFGAGAALQIPFAMQMAYRFNGIGVTQNVLYENQKLIA
HKU1          954 *SESQ*SG**TAATVAAMFPP*SAA----AG***SLNVQ**I**L***MD**NK******

SARS_CoV_2    925 NQFNSAIGKIQDSLSSTASALGKLQDVVNQNAQALNTLVKQLSSNFGAISSVLNDILSRL
HKU1         1010 TA**N*LLS**NGF*A*N***A*I*S***S******S*LQ**FNK******S*QE*****

SARS_CoV_2    985 DKVEAEVQIDRLITGRLQSLQTYVTQQLIRAAEIRASANLAATKMSECVLGQSKRVDFCG
HKU1         1070 *AL**Q*******N***TA*NA**S***SDISLVKFG*A**ME*VN***KS**P*IN***

SARS_CoV_2   1045 KGYHLMSFPQSAPHGVVFLHVTYVPAQEKNFTTAPAICHDGKAH-FPREGVFVSNGTHWF
HKU1         1130 N*N*IL*LV*N**Y*LL*M*FS*K*ISF*TVLVS*GL*IS*DVGIA*KQ*Y*IKHND**M

SARS_CoV_2   1104 VTQRNFYEPQIITTDNTFVSGNCDVVIGIVNNTVYDPLQPELDSFKEELDKYFKNHTSPD
HKU1         1190 F*GSSY*Y*EP*SDK*VVFMNT*S*NFTKAPLVYLNHSV*K*SD*ES**SHW***Q**IA

SARS_CoV_2   1164 VDLG-DISGINASVVNIQKEIDRLNEVAKNLNESLIDLQELGKYEQYIKWPWYIWLGFIA
HKU1         1250 PN*TLNLHT***TFLDLYY*MNLIQ*SI*S**N*Y*N*KDI*T**M*V*****V**LISF

SARS_CoV_2   1223 GLIAIVMVTIMLCCMTSCCS-CLKGCCSCGSCCKFDEDDSEPVLKGVKLHYT
HKU1         1310 SF*IFLVLLFFI**C*G*G*A*FSK---*HN**DEYGGHHDF*I*TSHD--D
```
