# Supplementary material for: Antibodies in serum of convalescent patients following mild COVID‐19 do not always prevent virus‐receptor binding
Source: Allergy. 2020 Aug 27;76(3):878–83. doi: 10.1111/all.14523 (PMC7984338; doi:10.1111/all.14523)
Supplement: Supplementary file 14 — Fig S13 [file ALL-76-878-s013.pdf]

FIGURE S13.

```
SARS_CoV_2     1 MFVFLVLLPLVSSQCVNLTTRTQLPPAYTNSFTRGVYYPDKVF-----------------
229E           1 ***L**AYA*LHIA--GCQ*TNG*NTS*SVC-NGC*G*SEN**AVESGGYIPSDFAFNNW

SARS_CoV_2    44 -----RSSVLHSTQDLFLPFFSNVTWFH----------AIHVSGTNGTKRFDNPVLPFND
229E          58 FLLTNT***VDGVVRS*Q*LLL*CL*SVSGLRFTTGFVYFNGT*RGDC*G*SSD**--S*

SARS_CoV_2    89 GVYFASTEKSNIIRGWIFGTTLDSKTQSLLIVNNATNVVIKVCEFQFCNDPFLGVYYHKN
229E         116 VIRYNLNFEE*LR**T*L-----F**SYGVV*FYC**NTLVSGDAHIPFGTV**NF*CFV

SARS_CoV_2   149 NKSWMESEFRVYSSANNCTFEYVSQPFLMDLEGKQGNFKNLREFVFKNIDGYFKIYSKHT
229E         171 *TTIGN----------ET*SAFVGA-----------LP*TVREFVISRT-*H*Y*N----

SARS_CoV_2   209 PINLVRDLPQGFSALEPLVDLPIGINITRFQTLLALH--RSYLTPGDSSSGWTAGAAAYY
229E         205 --------------------------GY*YF**GNVEAVNFNV*TAETTDFC*VAL*S*A

SARS_CoV_2   267 VGYLQPRTFLLKYNENGTITDAVDCALDPLSETKCTLKSFTVEKGIYQTSNFR-VQPTES
229E         239 --------DV*VNVSQTS*ANIIY*N-SVINRLR*DQL**D*PD*F*S**PIQS*ELPV*

SARS_CoV_2   326 IVRFPNITN------------------------------L----------CPFGEVFNAT
229E         290 **SL*VYHKHTFIVLYVDFKPQSGGGKCFNCYPAGVNIT*ANFNETKGPL*VDTSH*-T*

SARS_CoV_2   346 RFASVYAWNRKRISNCVADYSVLYNSASFSTFKCYGVSPTKLNDLCFTNVYADSFVIRGD
229E         349 KYVA***N-V------------GRW***IN*GN*P-F*FG*V*NF----------*----

SARS_CoV_2   406 EVRQIAPGQTGKIADYNYKLPDDFTGCVIAWNSNNLDSKVGGNYNYLYRLFRKSNLKPFE
229E         381 -----------*FGSVCFS*K*IPG**AMPIVA*WAY*----------------------

SARS_CoV_2   466 RDISTEIYQAGSTPCNGVEGFNCYFPLQSYGFQPTNGVGYQPYRVVVLSFELLHAPATVC
229E         408 ----------------------K*YTIG*LYVSWSD*D*------------ITGV*QP*E

SARS_CoV_2   526 GPKKSTNLVKNKCVNFNFNGLTGTGVLTESNKKFLPFQQFGRDIADTTDAVRDPQTLEIL
229E         434 *VSSFM*VTLD**TKY*IYDVS*V**IRV**DT**NGITYTSTSG-NLLGFK*VTKGT*Y

SARS_CoV_2   586 DITPCSFGGVSVITPGTNTSNQVAVLY---------------QDVNCTEVPVAIHADQLT
229E         493 S****NPP------------D*LV*YQQAVVGAMLSENFTSYGFS*VV*LPKFFY*S---

SARS_CoV_2   631 PTWRVYSTGSNVFQTRAGCLIGAEHVNNSYECDIPI----GAGICASYQTQTNSPRRARS
229E         538 --------------------------*GT*N*TDAVLTYSSF*V**DGSIIAVQ**NVSY

SARS_CoV_2   687 VASQSIIAYTMSLGAENSVAYSNNSIAIPTNFTISVTTEILPVSMTKTSVDCTMYICGDS
229E         572 DSVSA---------------IVTANLS**S*W*T**QV*Y*QITS*PIV***ST*V*NGN

SARS_CoV_2   747 TECSNLLLQYGSFCTQLNRALTGIAVEQDKNTQEVFAQVKQIYKTPPIKDFGGFNFSQIL
229E         617 VR*VE**K**T*A*KTIED**RNS*RLESADVS*MLTFD*KAFTLANVSS**DY*L*SVI

SARS_CoV_2   807 PD----PSKPSKRSFIEDLLFNKVTLADAGFIK-QYGDCLGDIAARDLICAQKFNGLTVL
229E         677 *SLPTSG*RVAG**A***I**S*LVTSGL*TVDAD*KK*TKGLSIA**A***YY**IM**

SARS_CoV_2   862 PPLLTDEMIAQYTSALLAGTITSGWTFGAGAALQIPFAMQMAYRFNGIGVTQNVLYENQK
229E         737 *GVADA*RM*M**GS*IG*IALG*L*----S*VS***SLAIQA*L*YVALQTD**Q****

SARS_CoV_2   922 LIANQFNSAIGKIQDS--------------LSSTASALGKLQDVVNQNAQALNTLVKQLS
229E         793 IL*AS**K*MTN*V*AFTGVNDAITQTSQA*QTV*T**N*I******QGNS**H*TS**R

SARS_CoV_2   968 SNFGAISSVLNDILSRLDKVEAEVQIDRLITGRLQSLQTYVTQQLIRAAEIRASANLAAT
229E         853 Q**Q****SIQA*YD***TIQ*DQ*V********AA*NVF*SHT*TKYT*V***RQ**QQ

SARS_CoV_2  1028 KMSECVLGQSKRVDFCGKGYHLMSFPQSAPHGVVFLHVTYVPAQEKNFTTAPAICHDGKA
229E         913 *VN***KS****YG***N*T*IF*IVNA**E*L****TVLL*T*Y*DVEAWSGL*V**TN

SARS_CoV_2  1088 HF-PREG--VFVSNGTHWFVTQRNFYEPQIITTDNTFVSGNCDVVIGIVNNTVYDPLQPE
229E         973 GYVL*QPNLALYKE*NYYRI*S*IMF**R*P*MADFVQIE**N*TFVNISRSELQTIV**

SARS_CoV_2  1145 L---DSFKEELDKYFKNHTSPDVDLGDISGINASVVNIQK-----------------EIDRLN
229E        1033 YIDVNKTLQ**SYKLP*Y*V**LVV---EQY*QTIL*LTSEISTLENKSAELNYTVQK*Q

SARS_CoV_2  1188 EVAKNLNESLIDLQELGKYEQYIKWPWYIWLGFIAGLIAIVMVTIMLCCMTSCCSC----
229E        1090 TLID*I*ST*V**KW*NRV*T******WV**CISVV**FV*SMLLLC**S*G**GFFSCF

SARS_CoV_2  1244 ---LKGCCSCGSCCKFDEDDSEPVLKGVKLHYT
229E        1150 ASSIR***ESTKLPYY*---------VE*I*IQ
```
