# Supplementary material for: Antibodies in serum of convalescent patients following mild COVID‐19 do not always prevent virus‐receptor binding
Source: Allergy. 2020 Aug 27;76(3):878–83. doi: 10.1111/all.14523 (PMC7984338; doi:10.1111/all.14523)
Supplement: Supplementary file 17 — Tab S1 [file ALL-76-878-s017.docx]

Table S1. Demographic and clinical characterization of subjects

| **Subject** | **gender^1^**  (f 11/m 14) | **age**  (range 18-70)  median 52.2 | **Time point serum obtained**  (month/year) | **Duration of symptoms^2^** [days] | **Symptoms^3^** | | | | | | | | | | | | | | | | |
| --- | --- | --- | --- | --- | --- | --- | --- | --- | --- | --- | --- | --- | --- | --- | --- | --- | --- | --- | --- | --- | --- |
|  |  |  |  |  | sneeze | rhinitis | conjunctivitis | arthralgia | myalgia | nausea | headache | chills | fever ^4^ | diarrhea | loss of smell /taste | Vertigo/ fatigue | Sore throat | cough | wheeze | Shortness of breath | pneumonia |
| B001 | f | 43 | 05/2020 | 19 | 1 | 1 | 1 | 0 | 0 | 0 | 0 | 1 | yes | 0 | 3 | 2 | 1 | 2 | 0 | 2 | 0 |
| B002 | f | 49 | 05/2020 | 17 | 1 | 1 | 1 | 0 | 0 | 1 | 3 | 0 | yes | 0 | 3 | 3 | 1 | 1 | 1 | 1 | 0 |
| B003 | m | 57 | 05/2020 | 10 | 1 | 0 | 0 | 0 | 1 | 0 | 2 | 2 | yes | 0 | 0 | 3 | 0 | 2 | 0 | 1 | 0 |
| B004 | m | 37 | 05/2020 | 10 | 0 | 0 | 0 | 2 | 0 | 0 | 3 | 1 | yes | 0 | 0 | 3 | 0 | 0 | 0 | 2 | 0 |
| B00X | f | 56 | 04/2020 | 11 | 1 | 0 | 0 | 1 | 3 | 0 | 3 | 2 | yes | 1 | 3 | 0 | 0 | 1 | 0 | 0 | 0 |
| B013 | m | 48 | 05/2020 | 14 | 0 | 0 | 0 | 1 | 1 | 0 | 1 | 0 | yes | 0 | 1 | 0 | 0 | 1 | 0 | 1 | 0 |
| B014 | f | 42 | 05/2020 | 5 | 1 | 3 | 0 | 1 | 1 | 0 | 1 | 0 | yes | 0 | 1 | 1 | 1 | 0 | 0 | 0 | 0 |
| B015 | m | 41 | 05/2020 | 14 | 0 | 2 | 0 | 0 | 3 | 2 | 0 | 0 | yes | 0 | 1 | 2 | 0 | 1 | 0 | 0 | 0 |
| B016 | m | 63 | 05/2020 | 5 | 0 | 0 | 0 | 3 | 0 | 0 | 2 | 0 | n.d | 0 | 0 | 0 | 0 | 0 | 0 | 0 | 0 |
| B017 | m | 60 | 05/2020 | 1 | 0 | 1 | 0 | 0 | 0 | 0 | 0 | 0 | yes | 0 | 0 | 0 | 0 | 0 | 0 | 0 | 0 |
| B018 | f | 70 | 05/2020 | 23 | 0 | 0 | 0 | 1 | 0 | 0 | 0 | 1 | yes | 0 | 0 | 3 | 2 | 3 | 0 | 2 | 0 |
| B019 | m | 70 | 05/2020 | 5 | 0 | 0 | 0 | 0 | 0 | 0 | 0 | 0 | n.d | 0 | 0 | 0 | 0 | 1 | 0 | 0 | 0 |
| B020 | f | 58 | 05/2020 | 21 | 0 | 0 | 1 | 3 | 3 | 2 | 3 | 3 | yes | 0 | 0 | 3 | 0 | 3 | 2 | 2 | 0 |
| B021 | m | 61 | 05/2020 | 13 | 0 | 2 | 0 | 1 | 1 | 0 | 3 | 3 | yes | 0 | 0 | 2 | 2 | 1 | 0 | 0 | 0 |
| B022 | m | 60 | 05/2020 | 21 | 0 | 0 | 0 | 1 | 2 | 1 | 0 | 2 | yes | 0 | 1 | 3 | 0 | 3 | 1 | 1 | 1 |
| B023 | f | 57 | 05/2020 | 20 | 2 | 0 | 0 | 1 | 0 | 0 | 0 | 1 | yes | 0 | 1 | 1 | 0 | 2 | 2 | 3 | 1 |
| B024 | f | 59 | 05/2020 | 19 | 0 | 1 | 0 | 0 | 2 | 0 | 1 | 1 | yes | 0 | 0 | 3 | 1 | 1 | 1 | 0 | 0 |
| B025 | f | 18 | 05/2020 | 14 | 2 | 2 | 1 | 0 | 0 | 0 | 1 | 0 | n.d | 3 | 3 | 3 | 2 | 3 | 0 | 0 | 0 |
| B026 | m | 33 | 05/2020 | 15 | 0 | 0 | 0 | 0 | 0 | 0 | 3 | 1 | yes | 0 | 3 | 3 | 0 | 1 | 0 | 0 | 0 |
| B027 | m | 28 | 05/2020 | 4 | 0 | 0 | 0 | 2 | 0 | 0 | 3 | 0 | yes | 0 | 0 | 2 | 0 | 0 | 0 | 0 | 0 |
| B028 | m | 78 | 05/2020 | 10 | 0 | 1 | 2 | 0 | 0 | 0 | 0 | 3 | yes | 0 | 1 | 3 | 1 | 2 | 0 | 1 | 0 |
| B029 | m | 67 | 05/2020 | 8 | 0 | 0 | 0 | 3 | 2 | 0 | 0 | 0 | yes | 0 | 3 | 0 | 0 | 1 | 0 | 0 | 0 |
| B030 | m | 58 | 05/2020 | 14 | 1 | 0 | 0 | 3 | 3 | 0 | 1 | 1 | yes | 0 | 1 | 3 | 0 | 3 | 0 | 0 | 0 |
| B031 | f | 56 | 05/2020 | 18 | 0 | 0 | 0 | 0 | 3 | 0 | 2 | 0 | yes | 2 | 3 | 1 | 0 | 1 | 0 | 1 | 0 |
| B032 | f | 38 | 05/2020 | 10 | 0 | 0 | 0 | 2 | 3 | 1 | 3 | 0 | n.d | 2 | 3 | 3 | 1 | 0 | 0 | 0 | 0 |

| **Subject** | **gender^1^**  (f 13/m 11) | **age**  (range 18-68)  median 43.2 | **Time point serum obtained**  (month/year) | **Duration of symptoms^2^ [days]** |
| --- | --- | --- | --- | --- |
| P001 | m | 18 | 01/2018 | n.a. |
| P002 | m | 36 | 04/2016 | n.a. |
| P003 | m | 29 | 01/2016 | n.a. |
| P004 | m | 38 | 05/2019 | n.a. |
| P005 | f | 28 | 05/2016 | n.a. |
| P006 | f | 28 | 04/2016 | n.a. |
| P007 | f | 39 | 04/2017 | n.a. |
| P008 | m | 56 | 10/2008 | n.a. |
| P009 | m | 69 | 04/2009 | n.a. |
| P010 | f | 60 | 05/2009 | n.a. |
| P011 | f | 43 | 04/2001 | n.a. |
| P012 | m | 24 | 04/2001 | n.a. |
| P013 | m | 47 | 05/2015 | n.a. |
| P014 | m | 49 | 04/2017 | n.a. |
| P015 | f | 43 | 10/2016 | n.a. |
| P016 | f | 33 | 10/2011 | n.a. |
| P017 | m | 61 | 10/2011 | n.a. |
| P018 | m | 59 | 11/2016 | n.a. |
| P019 | f | 42 | 06/1995 | n.a. |
| P020 | f | 50 | 12/2008 | n.a. |
| P021 | f | 45 | 06/1994 | n.a. |
| P022 | f | 51 | 05/1998 | n.a. |
| P023 | f | 40 | 04/2017 | n.a. |
| P00X | f | 50 | 11/2014 | n.a. |
|  |  |  |  |  |

^1^f = female, m = male

^2^ Duration of self-reported COVID-19 symptoms, n.a = not applicable

^3^ Self-reported symptoms: 0= none, 1= mild, 2= moderate, 3 =severe

^4^ Temperature >37.3 °C, n.d = not determined
