# Supplementary material for: Antibodies in serum of convalescent patients following mild COVID‐19 do not always prevent virus‐receptor binding
Source: Allergy. 2020 Aug 27;76(3):878–83. doi: 10.1111/all.14523 (PMC7984338; doi:10.1111/all.14523)
Supplement: Supplementary file 18 — Tab S2 [file ALL-76-878-s016.docx]

Table S2. SARS-CoV-2-specific antibodies and their effects on the RBD-ACE2 interaction

|  | **Direct ELISA** | | | | **Commercial test Siemens**^1^ | | **Interaction assay** | |
| --- | --- | --- | --- | --- | --- | --- | --- | --- |
| **Subject** | **IgG to S [OD]** | **IgG to RBD [OD]** | **IgM to S [OD]** | **IgM to RBD [OD]** | **[Index]** | **[RLU]** | **OD** | **% inhibition**^2^ |
| B001 | 1.90 | 2.87 | 1.35 | 2.48 | > 10,00 | 680389 | 2.08 | -18.3 |
| B002 | 2.01 | 2.86 | 0.97 | 1.03 | > 10,00 | 1265876 | 2.15 | -23.0 |
| B003 | 0.61 | 0.13 | 0.97 | 0.83 | 3.42 | 96867 | 1.36 | 29.4 |
| B004 | 1.20 | 1.00 | 0.80 | 0.25 | > 10,00 | 293965 | 2.43 | -41.6 |
| B00X | 0.92 | 0.04 | 0.81 | 0.71 | 0.56 | 25190 | 1.88 | -4.9 |
| B013 | 1.24 | 1.40 | 1.59 | 1.65 | > 10,00 | 535044 | 0.95 | 56.5 |
| B014 | 1.28 | 1.14 | 0.66 | 0.76 | > 10,00 | 352477 | 1.58 | 14.7 |
| B015 | 2.44 | 3.12 | 0.81 | 2.76 | > 10,00 | 1695729 | 1.10 | 47.0 |
| B016 | 0.77 | 0.33 | 0.78 | 0.72 | 2.08 | 64229 | 1.83 | -1.4 |
| B017 | 2.17 | 2.03 | 1.26 | 1.50 | > 10,00 | 1216125 | 0.91 | 59.5 |
| B018 | 1.99 | 3.40 | 0.61 | 1.60 | > 10,00 | 1157166 | 1.02 | 52.3 |
| B019 | 1.37 | 0.76 | 0.81 | 1.04 | > 10,00 | 273389 | 1.01 | 52.8 |
| B020 | 0.72 | 0.22 | 0.78 | 0.93 | 2.2 | 67381 | 1.39 | 27.7 |
| B021 | 0.97 | 0.99 | 1.64 | 2.49 | > 10,00 | 438565 | 1.77 | 2.5 |
| B022 | 1.80 | 2.56 | 0.98 | 1.57 | > 10,00 | 1191095 | 1.97 | -11.1 |
| B023 | 2.05 | 3.05 | 0.95 | 1.68 | > 10,00 | 1320357 | 1.93 | -8.4 |
| B024 | 0.85 | 1.10 | 1.25 | 0.78 | 4.92 | 132151 | 1.37 | 29.0 |
| B025 | 2.00 | 1.94 | 2.89 | 0.66 | > 10,00 | 778668 | 1.63 | 11.9 |
| B026 | 0.70 | 0.52 | 1.12 | 1.79 | 5.23 | 139164 | 2.03 | -15.0 |
| B027 | 0.98 | 0.91 | 1.01 | 0.46 | > 10,00 | 373981 | 1.60 | 13.3 |
| B028 | 2.37 | 3.37 | 0.81 | 0.85 | > 10,00 | 2072765 | 2.15 | -23.0 |
| B029 | 0.37 | 0.31 | 1.25 | 0.47 | 0.69 | 28589 | 0.69 | 73.8 |
| B030 | 1.77 | 2.55 | 2.89 | 3.13 | > 10,00 | 1048322 | 0.91 | 59.4 |
| B031 | 1.25 | 2.46 | 1.12 | 1.48 | > 10,00 | 891523 | 1.16 | 43.0 |
| B032 | 1.65 | 2.46 | 1.01 | 1.00 | > 10,00 | 1285956 | 1.28 | 34.8 |
| P001 | 0.03 | 0.01 | 0.97 | 0.49 | < 0,05 | 7284 | 1.87 | -4.6 |
| P002 | 0.02 | 0.02 | 0.67 | 0.07 | < 0,05 | 7781 | 1.89 | -5.7 |
| P003 | 0.05 | 0.02 | 0.16 | 0.16 | < 0,05 | 8831 | 1.88 | -4.8 |
| P004 | 0.07 | 0.01 | 0.04 | 0.07 | < 0,05 | 8398 | 1.80 | 0.3 |
| P005 | 0.07 | 0.01 | 0.04 | 0.10 | < 0,05 | 8865 | 1.67 | 9.3 |
| P006 | 0.06 | 0.01 | 0.04 | 0.49 | < 0,05 | 8026 | 1.80 | 0.3 |
| P007 | 0.25 | 0.05 | 0.47 | 0.07 | < 0,05 | 8862 | 1.79 | 1.1 |
| P008 | 0.69 | 0.05 | 0.65 | 0.18 | < 0,05 | 6743 | 1.74 | 4.1 |
| P009 | 0.07 | 0.01 | 0.69 | 0.51 | 0.1 | 11978 | 1.86 | -3.8 |
| P010 | 0.13 | 0.02 | 0.39 | 0.11 | < 0,05 | 7305 | 1.83 | -1.9 |
| P011 | 0.12 | 0.00 | 0.36 | -0.01 | < 0,05 | 7680 | 1.82 | -0.7 |
| P012 | 0.04 | 0.01 | 0.32 | 0.13 | < 0,05 | 8971 | 1.66 | 9.7 |
| P013 | 0.06 | 0.03 | 0.53 | 0.14 | 0.05 | 10326 | 1.75 | 3.7 |
| P014 | 0.32 | 0.16 | 0.90 | 1.42 | < 0,05 | 9897 | 2.37 | -37.7 |
| P015 | 0.04 | 0.02 | 0.56 | 0.35 | < 0,05 | 8579 | 1.83 | -1.8 |
| P016 | 0.16 | 0.01 | 0.56 | 0.54 | < 0,05 | 8204 | 1.62 | 12.4 |
| P017 | 0.22 | 0.02 | 0.64 | 0.34 | < 0,05 | 8310 | 1.83 | -1.3 |
| P018 | 0.08 | 0.01 | 0.26 | 0.06 | < 0,05 | 7323 | 1.66 | 9.7 |
| P019 | 0.06 | 0.01 | 0.55 | 0.08 | < 0,05 | 4861 | 1.82 | -0.8 |
| P020 | 0.04 | 0.01 | 0.66 | 0.12 | < 0,05 | 8562 | 1.69 | 7.6 |
| P021 | 0.09 | 0.03 | 0.34 | 0.67 | < 0,05 | 7195 | 1.65 | 10.5 |
| P022 | 0.07 | 0.02 | 0.58 | 0.05 | < 0,05 | 9044 | 1.79 | 1.4 |
| P023 | 0.05 | 0.01 | 0.29 | 0.02 | < 0,05 | 6974 | 1.89 | -5.7 |
| P00X | 0.09 | 0.01 | 0.77 | 0.10 | < 0,05 | 6440 | 1.83 | -1.3 |

^1^ Siemens, Atellica IM SARS-CoV-2 Total (COV2T). Index levels: <1 are negative, > 1 are considered positive. RLU: raw data of relative light units

^2^ Effects of antibodies on the RBD-ACE2 interaction: Moderate inhibition: >10 to 50 %; strong inhibition: > 50 %; enhancement: < -10%.
