# Supplementary material for: Antibodies in serum of convalescent patients following mild COVID‐19 do not always prevent virus‐receptor binding
Source: Allergy. 2020 Aug 27;76(3):878–83. doi: 10.1111/all.14523 (PMC7984338; doi:10.1111/all.14523)
Supplement: Supplementary file 19 — Tab S3 [file ALL-76-878-s002.docx]

Table S3.

| Peptide | aa sequence^1^ | Length [aa] | MW [Dalton]^2^ | pI^2^ |
| --- | --- | --- | --- | --- |
| 1 | PLVSSQCVNLTTRTQLPPAYTNSFTRGVYY | 30 | 3377,82 | 9,26 |
| 2 | RGVYYPDKVFRSSVLHSTQDLFLPFFSNVT | 30 | 3520,99 | 8,5 |
| 3 | FSNVTWFHAIHVSGTNGTKRFDNPVLPFND | 30 | 3418,78 | 6,92 |
| 4 | TLDSKTQSLLIVNNATNVVIKVCEFQFCND | 30 | 3357,84 | 4,56 |
| 5 | QFCNDPFLGVYYHKNNKSWMESEFRVYSSA | 30 | 3648,04 | 6,75 |
| 6 | VYSSANNCTFEYVSQPFLMDLEGKQGNFKN | 30 | 3431,8 | 4,68 |
| 7 | GNFKNLREFVFKNIDGYFKIYSKHTPINLV | 30 | 3603,18 | 9,7 |
| 8 | NITRFQTLLALHRSYLTPGDSSSGWTAGAA | 30 | 3192,54 | 8,75 |
| 9 | TAGAAAYYVGYLQPRTFLLKYNENGTITDA | 30 | 3282,66 | 5,73 |
| 10 | TITDAVDCALDPLSETKCTLKSFTVEKGIY | 30 | 3262,73 | 4,44 |
| 11 | EKGIYQTSNFRVQPTESIVRFPNITNLC | 28 | 3255,7 | 8,29 |
| 12 | PTESIVRF**PNITNLCPFGEVFNATR** | 25 | 2823,22 | 6,52 |
| 13 | **FNATRFASVYAWNRKRISNCVADYS** | 25 | 2940,29 | 9,78 |
| 14 | **VADYSVLYNSASFSTFKCYGVSPTK** | 25 | 2735,06 | 8,11 |
| 15 | **VSPTKLNDLCFTNVYADSFVIRGDEVRQIA** | 30 | 3371,81 | 4,68 |
| 16 | **VRQIAPGQTGKIADYNYKLPDDFTGCVIAW** | 30 | 3340,8 | 6 |
| 17 | **CVIAWNSNNLDSKVGGNYNYLYRLFRKSNL** | 30 | 3522,99 | 9,52 |
| 18 | **DSKVGGNYNYLYRLFRKSNLKPFER** | 25 | 3065,48 | 9,99 |
| 19 | **KPFERDISTEIYQAGSTPCNGVEGF** | 25 | 2746 | 4,41 |
| 20 | **GVEGFNCYFPLQSYGFQPTNGVGYQPYRVV** | 30 | 3387,77 | 5,99 |
| 21 | **PYRVVVLSFELLHAP**ATVCGPKKSTNLVKN | 30 | 3281,91 | 9,65 |
| 22 | PFQQFGRDIADTTDAVRDPQTLEILDIT | 28 | 3176,49 | 3,9 |
| 23 | AVLYQDVNCTEVPVAIHADQLTPTWRVYST | 30 | 3390,82 | 4,54 |
| 24 | RVYSTGSNVFQTRAGCLIGAEHVNNSYECD | 30 | 3291,57 | 5,44 |
| 25 | SYECDIPIGAGICASYQTQTNSPRRARSVA | 30 | 3215,56 | 7,78 |

^1^Amino acid sequence of synthesized peptides, SARS-CoV-2 RBD is highlighted in bold

^2^Molecular weight and pI were predicted by ExPASy ProtParam tool.
